# The evolving therapeutic landscape of gut-pancreatic peptide signalling in metabolic disorders: from mono- to multi-agonist therapies

**DOI:** 10.1042/BSR20250114

**Published:** 2026-07-16

**Authors:** Mohan Patil, Federico Piccinini, Elizabeth K.M. Johnstone, Ilaria Casari, Marco Falasca

**Affiliations:** 1 Molecular Endocrinology and Pharmacology, Harry Perkins Institute of Medical Research and Centre for Medical Research, The University of Western Australia, Nedlands, WA, Australia; 2 School of Biomedical Sciences, The University of Western Australia, Nedlands, WA, Australia; 3 Department of Medicine and Surgery, University of Parma, Parma 43125, Italy; 4 Lipovexa SRL, Via Giuseppe Mangionello, 12, 73024 Maglie (LE), Italy

**Keywords:** glucagon-like peptide-1, non alcoholic fatty liver disease, obesity, type 2 diabetes

## Abstract

The pharmacotherapeutic landscape for the clinical management of type-2 diabetes (T2D), obesity, metabolic dysfunction-associated steatotic liver disease, and steatohepatitis is evolving swiftly in response to the escalating global prevalence and incidence of these interrelated metabolic disorders. Although insulin and metformin formulations have long constituted the foundation of diabetes care, a paradigm shift in T2D management has been observed with the advent of novel pharmacotherapies. Gut peptide analogues are at the forefront of this transformation. The emergence of glucagon-like peptide-1 (GLP-1) receptor agonists represents a watershed moment, fundamentally reshaping the therapeutic landscape for both T2D and obesity due to multifaceted metabolic benefits. The clinical success of GLP-1-based therapies has stimulated pharmaceutical interest in other metabolic peptides. Gut-pancreatic peptides such as glucose-dependent insulinotropic polypeptide, glucagon, amylin, and peptide YY are of particular interest due to their distinct pharmacological benefits and therapeutic promise in metabolic disorders. The present review aims to provide a comprehensive and current overview of non-insulin gut-pancreatic peptide signalling-based therapies that are either clinically approved or under clinical investigation, with a focus on the emerging therapeutic convergence between T2D, obesity, and associated liver disease. The review critically narrates their mechanisms of action, therapeutic efficacy, limitations, current development status, and positioning in the treatment landscape. Furthermore, the review delineates the emerging avenues in the development of novel peptide-based pharmacotherapies, offering insights into their future potential and acquainting the reader with developments in non-insulin gut-pancreatic peptide signalling-based therapies for metabolic disorders.

## Introduction

Type-2 diabetes (T2D), obesity, metabolic dysfunction-associated steatotic liver disease (MASLD), and steatohepatitis (MASH) are interconnected metabolic disorders with shared intricate pathophysiology connections such as insulin resistance (IR), chronic low-grade inflammation, and dysregulated lipid homeostasis. These metabolic conditions often co-exist and significantly increase the risk of co-morbidities and mortality [1–5]. Their rising global prevalence poses a critical public health challenge [6–9].

Over the past decade, clinical management of diabetes has undergone a substantial transformation with the regulatory approval and clinical implementation of innovative pharmacological drug classes [10]. Among these, the principal incretin hormone, glucagon-like peptide-1 receptor agonist (GLP-1RA) therapies have gained prominence not only for their glycaemic and weight regulatory efficacy but also for their pleiotropic impacts on cardiovascular (CV) [11], renal [12], hepatic [13], and central nervous systems (CNS) [14], marking a shift towards comprehensive T2D management. Maintaining a healthy body weight and tight glycaemic control remains fundamental to both the prevention and treatment of metabolic conditions [10,15]. GLP-1RAs have demonstrated efficacy in achieving these clinical targets [16,17], thereby exerting beneficial effects in MASH. The weight regulation by GLP-1RAs predominantly arises from mechanisms including appetite suppression, modulation of central satiety pathways, and delayed gastric emptying [18]. Currently, several GLP-1RAs are in clinical practice [10], while numerous novel GLP-1 formulations are progressing through advanced stages of clinical development for metabolic indications, including T2D, obesity, and MASH.

**Figure 1:**
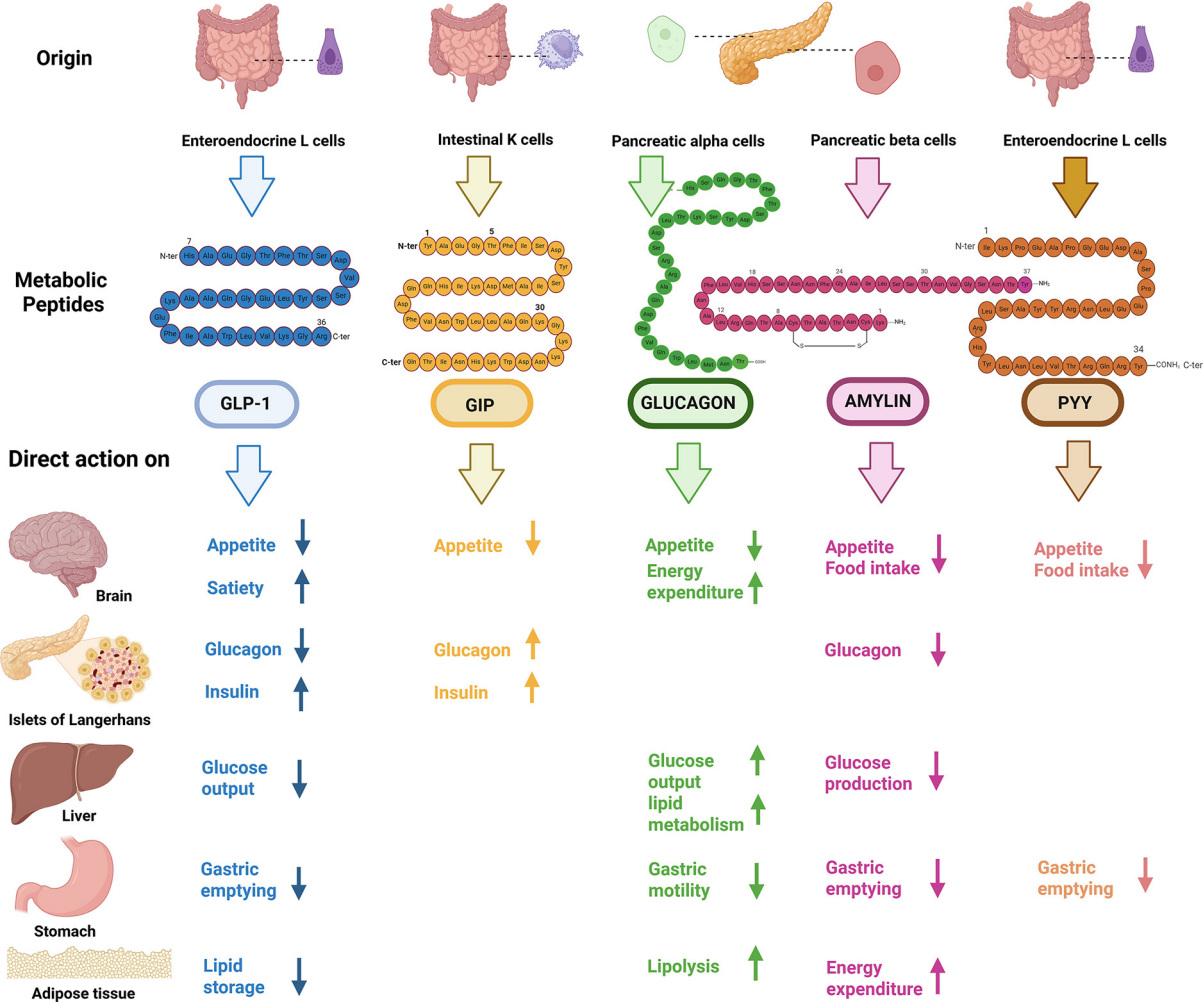
Metabolic Peptides of Pharmaceutical Interest in Metabolic Disorders: Origin, Structure, and Physiological Actions Metabolic peptides of growing pharmaceutical interest in metabolic disorders: origin, structure, and actions. Figure created with BioRender.com.

The multifaceted metabolic benefits conferred by GLP-1RAs have ignited pharmaceutical exploration aimed at achieving enhanced glycaemic control and more substantial body weight reduction. Consequently, the therapeutic potential of additional gut-pancreatic peptides is increasingly being investigated, either as monotherapy or in combination with GLP-1, to comprehensively target metabolic disorders [19]. Prominent gut-pancreatic peptide signalling currently under pharmacological investigation includes glucose-dependent insulinotropic polypeptide (GIP), amylin, peptide YY (PYY), and glucagon, owing to their unique and complementary metabolic benefits (Figure 1) [20]. In this context, combined agonism of GLP-1 and GIP receptors has demonstrated superior metabolic outcomes, notably enhanced glycaemic control, significant bodyweight reduction, and, consequently, promising hepatic benefits [21,22]. As a result, tirzepatide, a dual GLP-1/GIP receptor co-agonist, has secured regulatory approval for managing T2D and obesity [23,24]. Furthermore, triple peptide agonist strategies and other non-incretin peptide signalling-based therapies are also currently under active investigation for their potential to achieve superior metabolic outcomes.

In the present review, we present a comprehensive summary of the currently available gut-pancreatic hormone signalling-based pharmacotherapies, along with a detailed overview of recent advancements in gut-pancreatic peptide signalling-targeted therapeutic strategies for metabolic indications. It critically examines approved agents and emerging therapeutic candidates with a focus on their mechanistic rationale, current developmental status, clinical efficacy, and potential to address unmet clinical needs in metabolic disease management.

### GLP-1

**Figure 2:**
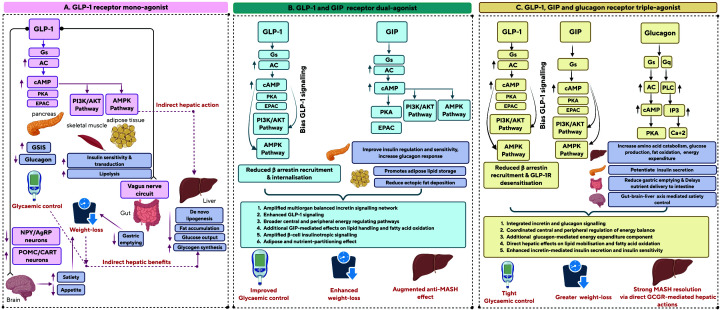
Schematic overview of the signalling architecture and metabolic consequences of mono-, dual-, and triple-incretin agonist therapies (**A**) GLP-1 mono-agonism predominantly activates G protein alpha-s (Gαs) signalling, increasing cAMP and engaging protein kinase A (PKA), exchange protein directly activated by cAMP (EPAC), phosphoinositide 3-kinase/protein kinase B (PI3K/AKT), and AMP-activated protein kinase (AMPK) pathways. These downstream signals enhance glucose-stimulated insulin secretion (GSIS), suppress glucagon secretion, promote satiety through hypothalamic neuropeptide Y/agouti-related peptide (NPY/AgRP) and pro-opiomelanocortin/cocaine-and amphetamine-regulated transcript (POMC/CART) neuronal circuits, delay gastric emptying, and indirectly improve hepatic glucose and lipid homeostasis. (**B**) Dual GLP-1/GIP agonism broadens incretin signalling by adding GIP-mediated Gαs-cAMP-PKA/EPAC, PI3K/AKT, and AMPK inputs, while reducing β-arrestin recruitment and receptor internalisation, consistent with biased GLP-1 receptor signalling. This expanded signalling network may augment β-cell insulinotropic activity, improve insulin sensitivity, and promote adipose nutrient partitioning and lipid storage with reduced ectopic fat deposition, thereby conferring greater metabolic efficacy than GLP-1 mono-agonism alone. (**C**) Triple GLP-1/GIP/glucagon agonism further extends the signalling repertoire through glucagon receptor (GCGR)-mediated Gαs and Gαq pathways, engaging AC/cAMP and phospholipase C (PLC)/inositol 1,4,5-trisphosphate (IP3)/intracellular calcium (Ca^+2^) signalling. These additional inputs provide direct hepatic actions that increase amino acid catabolism, hepatic glucose production, lipid mobilisation, and fatty acid oxidation and may increase energy expenditure. In parallel, triple agonism may potentiate insulin secretion and strengthen gut–brain–liver satiety signalling and delayed gastric emptying. Collectively, the progressive recruitment of complementary metabolic pathways provides a mechanistic basis for the broader and potentially additive benefits of dual and triple agonism on glycaemic control, weight loss, and MASH. Figure created with BioRender.com.

Human GLP-1 is a 30-residue peptide secreted by enteroendocrine L-cells (EEC) of the distal ileum and colon in response to nutrient ingestion. Following release, the native GLP-1 peptide undergoes rapid proteolytic cleavage by dipeptidyl peptidase-IV (DPP-IV), resulting in a short (<2 min) plasma half-life, which limits the therapeutic applicability of its unmodified form [18]. GLP-1 analogues are synthetic entities developed with structural modifications on the native GLP-1 peptide, enabling resistance to enzymatic degradation by DPP-IV, while retaining the ability to mimic endogenous GLP-1 actions. They regulate glucose and lipid homeostasis in T2D by enhancing glucose-stimulated insulin secretion, suppressing glucagon and hepatic glucose output, slowing gastric emptying, and reducing appetite [25]. The precise mechanisms by which GLP-1 analogues improve the pathophysiology of MASLD remain under active investigation. Although the presence of GLP-1 receptors in the liver is suggestive of direct hepatic actions [26], current evidence predominantly supports indirect actions mediated through weight reduction, improved hepatic glucose and lipid metabolism, and suppression of hepatic inflammation [27]. At the molecular level, GLP-1Rs are G protein-coupled receptors (GPCRs) whose activation stimulates adenylate cyclase (AC), elevating intracellular cyclic adenosine monophosphate (cAMP) and initiating downstream signalling cascades that promote glucose-dependent insulin secretion, inhibit glucagon release, delay gastric emptying, and reduce appetite, thereby integrating the regulation of glucose homeostasis and energy balance [28,29]. Beyond the pancreas, neuroendocrine integration along the gut–brain–liver axis has been defined, whereby intestinal GLP-1 activates vagal afferents projecting to the nucleus tractus solitarius (NTS) and hypothalamic nuclei that regulate appetite and hepatic glucose production (Figure 2A). This physio-pathological framework provides a mechanistic basis for the extra-pancreatic effects of GLP-1R agonists on body weight, lipid metabolism, and hepatic inflammation [30–32].

#### GLP-1 analogues in clinical use

*Exenatide*, the first short-acting GLP-1 analogue, was approved in 2005 for twice-daily subcutaneous (SC) treatment of T2D. Subsequent agents, including liraglutide (2010), lixisenatide (2013), dulaglutide (2014), and semaglutide (2017) marked the progressive expansion of this therapeutic class for the clinical management of T2D and obesity [33]. In this section, we focus on selected long-acting analogues currently in clinical practice and under trial for metabolic conditions.

#### Liraglutide

In 2014, Saxenda® (*liraglutide* 3 mg), administered once daily by SC injection, became the first GLP-1RA therapy approved for chronic weight management [34], complementing its established therapeutic indication for T2D as Victoza® (liraglutide 1.8 mg). Liraglutide is a long-acting GLP-1 analogue with 97% sequence identity to the human GLP-1 [7–37] peptide. It differs from native GLP-1 by a single amino acid substitution at position 34 (Lys->Arg) and by acylation of Lys26 with a C16 palmitic acid side chain *via* a glutamic acid spacer. This lipidation promotes strong, reversible albumin binding and resistance to DPP-IV-enzyme-mediated degradation and renal clearance, thereby enabling once-daily dosing [35].

The clinical efficacy of liraglutide has been extensively evaluated in randomised controlled trials within the LEADER and SCALE programmes for T2D and obesity, respectively [36–39]. In LEADER, liraglutide (1.8 mg daily) demonstrated potent anti-hyperglycaemic effects, significantly lowering glycated haemoglobin (HbA1c) and major adverse CV events (MACE) in patients with T2D. In parallel, the SCALE program demonstrated substantial and sustained weight loss with liraglutide (3 mg daily) in individuals with obesity, irrespective of diabetic status [36–38]. However, long-term follow-up in the SCALE study (NCT01272219) demonstrated weight regain in patients receiving liraglutide after treatment discontinuation [39].

Evidence for hepatic benefits emerged from the LEAN trial (NCT01237119), in which 52 adults with biopsy-confirmed non-alcoholic steatohepatitis (NASH, currently MASH) and body mass index (BMI) ≥25 kg/m^2^ received liraglutide 1.8 mg daily for 48 weeks. MASH resolution without fibrosis progression occurred in 39% of liraglutide-treated participants versus (vs) 9% in the placebo group [40], providing the first histological evidence that a GLP-1RA may promote MASH resolution under controlled metabolic conditions. Imaging-based trials have reinforced these observations, as seen in the LIRAINS study (NCT01399645), in which adults with T2D and MASLD received liraglutide (0.6–1.8 mg daily) for 12 weeks and exhibited superior reductions in hepatic fat content than those receiving insulin glargine [41]. A six-month prospective cohort further reported a 31% relative reduction in liver fat with liraglutide 1.2 mg/day, correlating with weight loss and improved glycaemic control [42]. Additionally, a 26-week randomised trial (NCT02147925) in 75 patients with T2D confirmed liraglutide’s superiority over insulin, reducing hepatic fat and visceral adiposity [43].

Collectively, these studies provide convergent evidence of histological improvement and sustained attenuation of hepatic steatosis, supporting GLP-1 agonism as a viable therapeutic target for metabolic liver disease. Following liraglutide, several additional GLP-1 analogues, including albiglutide (2014; discontinued in 2017) [44], dulaglutide (2014) [45], lixisenatide (2016; discontinued in 2023), and semaglutide (2017), received regulatory approval. Among these agents, semaglutide has shown particularly robust and sustained clinical adoption, building upon the therapeutic framework established by liraglutide.

#### Semaglutide

*Semaglutide* is a long-acting GLP-1 analogue sharing 94% sequence homology with the native peptide, engineered through targeted structural modifications to enhance metabolic stability and prolong pharmacokinetics (PK). These include substitution of alanine at position 8 with α-aminoisobutyric acid, conferring resistance to DPP-IV-mediated degradation, and conjugation of a C18 fatty acid *via* a spacer that promotes reversible albumin binding and renal clearance and extends the elimination half-life (*t*_1/2_), thereby enabling once-weekly dosing [46]. Consequently, semaglutide exhibits robust efficacy in maintaining tight glycaemic control, reducing body weight, and lowering CV and renal risk [28]. Head-to-head trials have demonstrated that semaglutide produces greater reductions in HbA1c and body weight than other GLP-1 receptor agonists, including exenatide (SUSTAIN 3), dulaglutide (SUSTAIN 7), and liraglutide (SUSTAIN 10), while maintaining a favourable safety profile predominantly characterised by mild-to-moderate, transient gastrointestinal (GI) adverse events.

Consistent improvements have also been observed across cardiometabolic parameters, particularly in systolic blood pressure (approximately 5–6 mmHg) and triglyceride concentrations (around 18%); these benefits were sustained with continued therapy but attenuated following transition to placebo [28,47]. Beyond metabolic control, GLP-1RAs confer significant CV and renal protection in individuals with T2D. In the FLOW trial, semaglutide 1 mg once weekly reduced the risk of the primary composite renal-CV endpoint by 24%, comprising kidney failure, a persistent 50% decline in eGFR, or death from kidney-related or CV causes [48]. The SUSTAIN-8 trial further demonstrated that, compared with the sodium-glucose cotransporter-2 inhibitor canagliflozin, semaglutide induced greater reductions in total and visceral fat mass, a proportional increase in lean mass, and improvements in quality of life, underscoring its favourable impact on body composition [28,49]. Notably, in individuals with overweight or obesity and CV disease but without diabetes, semaglutide 2.4 mg was associated with a 20% reduction in major CV events (CV death, nonfatal myocardial infarction, or nonfatal stroke), accompanied by sustained long-term weight loss [50]. However, in the STEP trials (NCT03548935 and NCT03548987), discontinuation of treatment was associated with a regain of approximately two-thirds of the weight previously lost, together with concomitant worsening of cardiometabolic parameters [51].

Clinical experience with semaglutide has driven the development of next-generation incretin-based therapies that combine GLP-1R agonism with additional hormonal targets, such as GIP or glucagon, to amplify effects on body weight and cardiometabolic risk [28,29]. In this context, the SURMOUNT-5 trial in individuals with obesity without diabetes employed semaglutide as the active comparator against tirzepatide, a dual GLP-1/GIPR agonist, and demonstrated a greater reduction in body weight (-20.2% vs -13.7%) and waist circumference (-18.4 cm vs -13.0 cm) with tirzepatide after 72 weeks, with comparable GI tolerability during dose escalation. While confirming the superiority of the dual agonism, these findings reinforce semaglutide’s role as the clinical benchmark within the mono GLP-1RA class [52].

Beyond its once-weekly SC injection formulation, semaglutide is also available as an oral preparation using the absorption enhancer SNAC (sodium N-[8-(2-hydroxybenzoyl] amino) caprylate). However, its low bioavailability (approximately 1%) and the requirement for strict fasting and water-intake conditions limit absorption consistency, contributing to the more reliable clinical efficacy observed with injection formulation [29,53]. Comparative analyses within the GLP-1RAs class indicate that semaglutide achieves greater reductions in HbA1c and weight than dulaglutide in head-to-head trials [54], and more pronounced weight loss than liraglutide in direct comparisons (-15.8% vs -6.4%) [55], while maintaining a consistent safety profile with limited severity GI adverse events; cholelithiasis and acute kidney injury remain infrequent, and overall safety data for the class are reassuring [54].

In metabolic liver disease, particular attention is focused on MASH. The Phase-3 ESSENCE trial is evaluating semaglutide 2.4 mg once-weekly in participants with MASH patients with F2–F3 stage fibrosis. The design and baseline analysis of the first 800 randomised participants revealed an exceptionally high prevalence of cardiometabolic risk features (>99% meeting at least one criterion) and defined two co-primary histological endpoints at 72 weeks: resolution of steatohepatitis without fibrosis progression and improvement of fibrosis without steatohepatitis worsening [56]. This trial design is supported by Phase-2 data in MASH with F1–F3 fibrosis, demonstrating a higher rate of steatohepatitis resolution with semaglutide than placebo, along with dose-dependent improvements in cardiometabolic parameters, although definitive effects on fibrosis require larger and longer-duration studies [56].

### Emerging injectable GLP-1 therapies

#### Efpeglenatide

*Efpeglenatide* (Hanmi Pharmaceutical, South Korea; licensed to Sanofi, France) is a long-acting GLP-1R agonist derived from exendin, modified by one amino acid and linked to the Fc region of human IgG4 through a 3.4 kDa mini-polyethylene glycol space. The Fc-conjugation gives it prolonged activity and a PK profile that supports flexible dosing intervals (weekly, biweekly, or monthly) [57]. In the Phase-2 EXCEED 203 trial (NCT02057172), 254 adults with early-stage T2D receiving metformin monotherapy were randomised to SC efpeglenatide 0.3, 1, 2, 3, or 4 mg once weekly; placebo; or liraglutide 1.8 mg once daily for 12 weeks. Relative to placebo, efpeglenatide at all doses ≥1 mg produced statistically significant reductions in HbA1c (placebo-adjusted least-squares mean reductions of 0.6%–1.2%), with 61%–72% of patients achieving HbA1c <7% vs 24% in the placebo group. The 3 and 4 mg regimens reduced body weight by 1.4 and 2.0 kg, respectively, compared with placebo. The 4 mg regimen demonstrated non-inferiority to liraglutide in HbA1c lowering [58]. In another Phase-2 LIBERATE 204 study (NCT02081118; *n* = 209), metformin-treated patients with T2D received efpeglenatide 8, 12, or 16 mg once monthly after a 4-week 4 mg once-weekly titration and a single 8 mg monthly lead-in versus placebo for 16 weeks. Efpeglenatide at all regimens improved glycaemic control, with least squares mean HbA1c reductions vs placebo of 0.66%, 0.67%, and 0.79%, respectively. Nearly 48.7% of efpeglenatide-treated patients achieved HbA1c < 7% compared with 30.6% in the placebo group, accompanied by a placebo-adjusted weight reduction of 2.0 kg [59]. Furthermore, in the Phase-2 BALANCE trial (NCT02075281; *n* = 297), non-diabetic adults with obesity or overweight and comorbidity received efpeglenatide 4 or 6 mg once weekly or 6 or 8 mg once every 2 weeks for 20 weeks with a hypocaloric diet restriction. The study met its primary efficacy objective, demonstrating placebo-adjusted body weight reductions of approximately 6.3–7.2 kg across all regimens, along with improvements in secondary metabolic outcomes, including the proportions of patients achieving ≥5% and ≥10% weight loss, as well as HbA1c, fasting glucose, and cholesterol and triglyceride levels [60]. Glycaemic and weight-lowering efficacy was corroborated in the Phase-3 AMPLITUDE-M trial (NCT03353350), in which 406 treatment-naive adults with T2D inadequately controlled with diet and exercise received once-weekly efpeglenatide at doses of 2, 4, and 6 mg for 56 weeks, with the primary endpoint defined as HbA1c change at week 30. Efpeglenatide treatment produced marked reductions in HbA1c from a baseline of 8.1% to 6.9%, 6.6%, and 6.4% with the 2, 4, and 6 mg regimens, respectively, corresponding to placebo-adjusted least squares mean differences of -0.5%, -0.8%, and -1.0%, and 60%–74% of patients achieved HbA1c < 7%. Fasting plasma glucose and body weight also declined significantly at the 4 and 6 mg doses at week 30 [61]. In the AMPLITUDE-O (Phase-3 CV outcomes trial; NCT03496298), 4076 patients with T2D and either established CV disease or chronic kidney disease plus an additional CV risk factor were randomised to efpeglenatide 4 mg or 6 mg once weekly or placebo for a median follow-up of 1.81 years. The primary endpoint, first major adverse CV event (nonfatal myocardial infarction, nonfatal stroke, or death from CV or undetermined causes), occurred in 7.0% of efpeglenatide-treated patients versus 9.2% with placebo (Hazard ratio (HR) 0.73, 95% (confidence interval (CI) 0.58–0.92). The key renal composite secondary endpoint occurred in 13.0 versus 18.4%, respectively (HR 0.68, 95% CI 0.57–0.79). Efpeglenatide also improved metabolic parameters, with greater reductions in HbA1c (-1.24%) and body weight (-2.6 kg) than placebo, together with a modest reduction in systolic blood pressure (approximately 1.4–1.6 mmHg) and a small increase in heart rate (approximately 3.5–4.3 beats/min). A subsequent dose-response analysis suggested that the 6 mg dose conferred greater protection than 4 mg for MACE and the major cardiorenal composite outcomes [62]. Across all clinical studies, transient GI adverse events were commonly observed with efpeglenatide treatment.

#### Noiiglutide

*Noiiglutide* (Jiangsu Hengrui Pharmaceuticals, China) is a once-daily, SC, DPP-IV-resistant GLP-1 analogue engineered through palmitic acid modification to confer *t*_1/2_ of approximately 10–12 h. It is currently being evaluated in a multicentre, randomised, double-blind Phase-3 trial (NCT06649773) to assess glycaemic efficacy over 24 weeks in adults with T2D inadequately controlled on metformin, with or without an additional oral antihyperglycemic agent. Earlier Phase-1 development (CTR20201346) established the safety, PK, and pharmacodynamic (PD) profile of noiiglutide in 40 Chinese adults with obesity (BMI ≥28 kg/m^2^). Using stepwise titration over 3–6 weeks, participants received maintenance doses of 0.18, 0.24, 0.30, or 0.36 mg/day. The study reported exploratory mean body-weight reductions of -3.26, -5.45, -4.35, and -7.46 kg across the ascending dose cohorts, compared with -1.89 kg with placebo [63]. In a subsequent Phase-2 trial (NCT04799327), noiiglutide was evaluated in 254 Chinese adults with obesity without diabetes (BMI 28.0–40.0 kg/m^2^) over 24 weeks. Participants were randomised to once-daily SC noiiglutide 0.12, 0.24, or 0.36 mg, or placebo (1:1:1:1). In the present study, noiiglutide reduced body weight by 8.03–8.50 kg vs 3.65 kg with placebo, with ≥5% weight loss achieved by 60.0%−73.0% of participants and ≥10% weight loss by 36.9%–39.7%, compared with 29.0% and 8.1%, respectively, in the placebo group. Nausea, diarrhoea, and vomiting were the most common adverse events and occurred more frequently than with placebo, consistent with the expected GLP-1RA safety profile [64]. Additionally, a fixed-ratio combination of basal insulin INS068 with noiiglutide was evaluated for 26 weeks in a Phase-2 study in 455 Chinese adults with T2D inadequately controlled on metformin, with or without a second oral antidiabetic drug. The combination achieved greater HbA1c reduction than INS068 or noiiglutide alone (-2.4% vs -1.5% and -1.7%), with more participants reaching HbA1c < 7.0% (81.4% vs 47.8% vs 55.6%) and ≤6.5% (74.3% vs 26.4% vs 41.1%), respectively [65].

#### TG103

*TG103* (CSPC Baike/Shijiazhuang Pharmaceutical Group, China) is a long-acting recombinant GLP-1-Fc fusion protein currently in Phase-3 clinical development for T2D and weight management. Confirmatory evidence is being sought in an ongoing multicentred, randomised, double-blind, placebo-controlled Phase-3 trial (NCT05997576) in non-diabetic overweight (BMI >24 to ≤28 kg/m^2^) or obese (BMI ≤28 kg/m^2^) adults with at least one obesity-related complication. This study is designed to evaluate once-weekly SC administration, with dose escalation from 7.5 mg to 22.5 mg, in conjunction with lifestyle intervention over 52 weeks. Earlier, in a Phase-1 single-ascending-dose study (NCT03990090), 32 healthy Chinese adults received a single SC dose of TG103 (3, 7.5, 15, or 22.5 mg) or placebo to characterise safety, tolerability, and PK/PD. TG103 exhibited a prolonged mean terminal half-life (*t*_1/2_) of 147.16–184.72 h and produced reductions in fasting blood glucose across all active-dose cohorts, most notably at the 15 mg dose. Treatment-related adverse events included decreased appetite (41.7%); nausea; flatulence; elevated urinary β2-microglobulin; increased serum total bile acid (20.8%); decreased high-density lipoprotein (16.7%); and abdominal distension (12.5%) [66,67]. These findings were extended in the Phase-1b multiple-dose trial (NCT04855292; *n* = 48), in which adults with overweight or obesity without diabetes (BMI ≥26.0 kg/m^2^; body weight ≥ 60 kg) received once-weekly therapy for 12 weeks, initiated at 7.5 mg and escalated at weekly intervals to 15.0, 22.5, or 30.0 mg, without mandated lifestyle intervention. At week 12, mean body-weight reductions were -5.65, -5.35, and -5.13 kg with 15, 22.5, and 30 mg, respectively, versus -1.37 kg with placebo. TG103 also resulted in numerical cardiometabolic improvements with mean systolic blood-pressure changes of -0.3, -5.6, and -16.1 mmHg versus +2.5 mmHg with placebo and diastolic blood pressure changed by -2.6, -0.7, and -4.1 mmHg versus +2.8 mmHg with placebo [66,67]. Importantly, despite progression into Phase-2 development across obesity and T2D populations (NCT05063253, NCT05299697, NCT05348122, and NCT06226090), efficacy and safety data from these studies remain unavailable in the public domain at the time of writing.

#### Ecnoglutide

*Ecnoglutide* (Sciwind Biosciences, China) is a cAMP-biased, long-acting GLP-1 analogue composed entirely of natural amino acid residues and incorporating site-specific fatty acid acylation to enhance albumin binding, prolong systemic exposure, improve DPP-IV resistance, and support once-weekly SC administration [68]. In a randomised, double-blind Phase-2 trial (CTR20211014), 145 adults with T2D inadequately controlled with diet and exercise alone or a single oral glucose-lowering agent were randomised 1:1:1:1 to once-weekly ecnoglutide (0.4, 0.8, or 1.2 mg) for 20 weeks. The primary endpoint, change in HbA1c at week 20, was achieved with placebo-adjusted HbA1c reductions of -26%, -1.35%, and -4%. Secondary endpoints were directionally concordant, with placebo-adjusted reductions in fasting blood glucose of -2.0, -1.9 and -2.7 mmol/l; body-weight reductions of -1.57 to -2.26 kg vs a +0.50 kg gain with placebo; and at least 5% weight loss in up to 33.3 versus 3.0%, respectively [69]. These findings were extended in EECOH-2, a 52-week, open-label, active-controlled, randomised Phase-3 trial (NCT05680129) in 623 adults with T2D inadequately controlled on metformin monotherapy, in which participants received once-weekly SC ecnoglutide (0.6 and 1.2 mg) or dulaglutide 1.5 mg. The study set the primary endpoint as the change in HbA1c at week 32, with non-inferiority prespecified for both ecnoglutide doses and superiority prespecified for the 1.2 mg dose. Mean HbA1c reductions at week 32 were -1.91% (0.6 mg), -1.89% (1.2 mg), and -1.65% with dulaglutide; the estimated treatment differences vs dulaglutide were -6% (95% CI -0.39 to -0.13) and -0.24% (95% CI -0.38 to -0.11), establishing non-inferiority for both doses and statistical superiority for 1.2 mg. HbA1c reductions were sustained through week 52. Reported secondary endpoints also favoured treatment, including glycaemic target attainment and improvements in fasting plasma glucose, 2-h postprandial glucose, body weight, waist and hip circumference, triglycerides, and BMI. Discontinuation because of adverse events occurred in 3%, 4%, and 3% of the ecnoglutide 0.6 mg, ecnoglutide 1.2 mg, and dulaglutide groups, respectively, and overall tolerability was consistent with the GLP-1RA class [70]. Across clinical studies, ecnoglutide was generally well tolerated, with no treatment-related grade ≥3 adverse events, serious adverse events, or deaths.

#### Utreglutide

*Utreglutide* (Sun Pharmaceutical Industries Limited, India) is a long-acting GLP-1 analogue formulated for once-weekly SC administration and is currently undergoing Phase-2 clinical development for obesity and T2D, with additional explorations in MASLD. Pharmacologically, utreglutide exhibits potency-driven G-protein bias, with signalling preferentially directed toward cAMP generation rather than GLP-1R endocytosis and β-arrestin-2 recruitment, thereby supporting differentiated metabolic pharmacology. Structurally, the molecule incorporates an Ala2->Aib substitution to confer DPP-IV resistance, a Lys28->Arg substitution to enable site-specific acylation at Lys20, a distinct linker conjugated to an octadecanedioic-acid albumin-binding moiety to prolong half-life, and an additional C-terminal leucine to enhance anti-diabetic and weight-reducing efficacy [71]. In a Phase-1 study (EudraCT No. 2020-003765-20) involving adults with overweight or obesity (BMI 28–45 kg/m^2^), 10 weeks of dose escalation (0.4 mg once, 0.8 mg twice, 1.4 mg three times, and 2.0 mg four times) resulted in a mean body weight change of -5.9 ± 3.2 kg (-5.9%) vs +0.3 ± 0.9 kg with placebo. Treatment was also associated with reductions in LDL cholesterol (-9.6%), ApoB (-16.3%), ALT (-22.2%), AST (-17.9%), GGT (-23.9%), and fatty liver index (77.6 ± 15.4 to 62.1 ± 24.8) [72]. Furthermore, in a Phase-1b/2a study (ACTRN12624000322538) of 22 postmenopausal obese women (BMI ≥30 kg/m^2^ with liver fat content ≥10%), utreglutide achieved an -8.0% body-weight reduction at Week 14, sustained through Week 17. Moreover, 76% and 71% of treated participants attained >5% weight loss at Weeks 14 and 17, respectively. Liver fat decreased by -28.6% (2.7% with placebo). About 35% of patients achieved >30% relative liver fat reduction. These changes coincided with improvements in the blood fibrosis marker, Pro-C3 (18.1 ng/ml), and leptin levels. Treatment-emergent adverse events included non-severe nausea, dyspepsia, and vomiting [73].

### Emerging oral GLP-1 formulations

Several novel oral small-molecule GLP-1RA formulations are in advanced clinical development, with the aim of addressing the practical and adherence limitations associated with injectable formulations.

#### Orforglipron

*Orforglipron* (Eli Lilly and Company, U.S.A.) is an orally active, non-peptide, small-molecule GLP-1RA developed for T2D and obesity. In a 26-week, Phase-2 trial (NCT05048719; *n* = 383), adults with T2D received once-daily orforglipron (3, 12, 24, 36, or 45 mg) or dulaglutide 1.5 mg weekly. Orforglipron produced mean HbA1c reductions of -1.24% to -2.10% compared with 0.43% with placebo and -1.10% with dulaglutide, corresponding to placebo-adjusted changes of -0.8% to -1.7%. Doses ≥12 mg also significantly lowered fasting glucose and body weight, with a maximal mean weight loss of -10.1 kg (95% CI, -11.5 to -8.7) compared with -2.2 kg for placebo and 3.9 kg for dulaglutide. Treatment-related GI events (44.1%–70.4%) were mostly transient in nature [74]. These observations were confirmed in ACHIEVE-1 (NCT05971940), a Phase-3 trial involving 559 adults with early T2D (mean baseline HbA1c 8.0%) treated with once-daily orforglipron 3, 12, or 36 mg or placebo for 40 weeks. Orforglipron treatment exhibited mean HbA1c reductions of -1.24%, -1.47%, and -1.48% compared with -0.41% in placebo; yielding placebo-adjusted differences of -0.83% (95% CI: -1.10 to -0.56), -1.06% (95% CI: -1.33 to -0.79), and -1.07% (95% CI: -1.33 to -0.81), respectively. Orforglipron also resulted in weight loss of -4.5%, -5.8%, and -7.6% versus -1.7% in the placebo group. GI adverse events were common and led to treatment discontinuation in 4.4%–7.8% of orforglipron-treated participants compared with 1.4% in the placebo group [75]. In parallel, Phase-3 ATTAIN-1 trial (NCT05872620; *n* = 3127) evaluated 72 weeks of once-daily orforglipron (6, 12, or 36 mg) in adults with obesity but without diabetes. The mean body weight change from baseline to week 72 was -7.5% (95% CI: -8.2 to -6.8) with 6 mg of orforglipron, -8.4% (95% CI: -9.1 to -7.7) with 12 mg, and -11.2% (95% CI: -12.0 to -10.4) with 36 mg, compared with -2.1% (95% CI: -2.8 to -1.4) with placebo. In the 36 mg group, 54.6% of participants achieved a ≥10% weight reduction; 36.0% ≥15%; and 18.4%, ≥20%, versus 12.9%, 5.9%, and 2.8%, respectively, in the placebo group. Treatment discontinuation due to GI adverse events occurred in 5.3%–10.3% versus 2.7% in the placebo arm [76]. In a subsequent 72-week ATTAIN-2 study (NCT05872620; *n* = 1613) in participants with a BMI of ≥27 kg/m^2^ and HbA1c of 7%–10%, once-daily oral orforglipron (6, 12, or 36 mg) significantly reduced body weight at week 72 by -5.1%, -7.0%, and -9.6%, respectively, compared with -2.5% with placebo. Secondary endpoints were also improved, including achievement of ≥5% weight loss (47.7%, 54.6%, 67.2% versus 26.6%), ≥10% weight loss (22.6%, 31.2%, 45.6% versus -9.0%), HbA1c reduction (-1.22%, -1.50%, -1.66% versus -0.47%), and attainment of HbA1c <7.0% (64.6%, 75.9%, 75.5% versus 30.5%) and ≤6.5% (52.5%, 57.6%, 66.6% versus 15.4%). Fasting serum glucose decreased by -30.5 to -42.4 mg/dl versus -9.3 mg/dl with placebo; at 36 weeks, waist circumference decreased by -8.3 versus -2.8 cm, with pooled reductions in systolic blood pressure (-4.2 vs -1.6 mm Hg) and triglycerides (-16.0% vs -4.2%). GI events led to discontinuation in 6.1%, 10.6%, and 10.6% of orforglipron groups vs 4.6% with placebo [77]. In April 2026, orforglipron (Foundayo™) was approved by the U.S. Food and Drug Administration (FDA) for chronic weight management in adults with obesity or overweight with at least one weight-related comorbidity, while regulatory applications in other jurisdictions remained under review at the time of writing.

#### Aleniglipron

*Aleniglipron* (Structure Therapeutics, U.S.A.) is another once-daily, oral small-molecule GLP-1RA with biased agonism and reduced β-arrestin signalling. A Phase-1b, 28-day, multiple ascending dose evaluation on healthy overweight or obese adults reported up to 4.9% placebo-adjusted body weight reduction. A subsequent Phase-2a trial (NCT05762471) in T2D showed placebo-adjusted HbA1c reductions of -1.01% to -1.02% and weight reductions of -3.26% to -3.51% at week 12. In parallel, a 12-week obesity study (*n* = 64, 120 mg) achieved a 6.2% placebo-adjusted mean weight loss. The 36-week Phase-2b ACCESS trial (NCT06693843; *n* = 230) demonstrated placebo-adjusted weight losses of -8.2%, -9.8%, and -11.3% for 45, 90, and 120 mg alenigripon, with concurrent HbA1c improvements of -0.28% to -0.37%. In the exploratory ACCESS II study (NCT06703021; *n* = 85), weight loss reached -15.3% at 240 mg [78].

#### CT-996

*CT-996* (Carmot Therapeutics Inc., U.S.A. and Roche, Switzerland) is an orthostatic GLP-1RA engineered with signalling bias, preferentially stimulating cAMP production with minimal β-arrestin recruitment [79]. A Phase-1 (NCT05814107) evaluation in overweight or obese adults, including some with T2D, reported CT-966 as well-tolerated, showing mainly low-grade GI adverse events with PK assessments (*T*_max_ 8–9.6 h; *t*_1/2_ 17.1–21.8 h) supporting once-daily dosing. In this dose titration study, CT-996 resulted in approximately a 7.3% reduction in mean body weight compared with placebo [80].

### GIP

Over the past decade, the pharmacology of GIP peptide has undergone a paradigm shift, with accumulating evidence demonstrating its therapeutic benefits, particularly through its incorporation as either an agonist or antagonist in dual and triple incretins. Native GIP peptide belongs to the secretin/vasoactive intestinal peptide family, consisting of a chain of 42-amino acid sequences, predominantly secreted by enteroendocrine K-cells in the duodenum and jejunum, primarily in response to dietary carbohydrates and lipids. Like GLP-1, activity of GIP is also believed to be limited by DPP-IV, resulting in a plasma half-life of approximately 5–7 min [81]. GIP orchestrates energy metabolism *via* glucose-stimulated insulin secretion and nutrient partitioning, exerting both insulin-dependent and insulin-mimetic effects [81]. In adipose tissue, it activates the PKB/LKB1/AMPK pathway, up-regulates lipoprotein lipase activity, and enhances lipogenesis via the adipokine-mediated resistin pathway [81,82]. Its overexpression in obesity is believed to foster insulin resistance due to hypersecretion [83]. The exaggerated postprandial GIP secretion initiates a ‘vicious cycle’ of enhanced nutrient uptake into adipocytes, aggravating insulin resistance, promoting hyperinsulinemia, and accelerating lipid deposition, thereby sustaining metabolic dysfunction [83].

This pathophysiological paradigm explains why GIP, despite its favourable insulinotropic and anabolic effects under euglycaemic conditions, was historically deemed an untenable therapeutic target in T2D. GIP resistance was first demonstrated using hyperglycaemic clamp studies, which showed a markedly reduced insulinotropic response in individuals with T2D, in contrast with the preserved efficacy of GLP-1 [81,84]. Subsequent work reported that GIP resistance reflects chronic hyperglycaemia-induced down-regulation of GIP receptor (GIPR) expression and enhanced receptor degradation via ubiquitination mechanisms, rather than an intrinsic defect of the peptide itself [85]. Recently, it has been discovered that the co-activation of GIP and GLP-1 receptors can overcome GIP resistance, restore physiological insulin secretion, and improve overall insulin sensitivity [86,87]. Furthermore, simultaneous receptor stimulation produces an additive effect on insulin secretion and glucose tolerance (Figure 2B). As both receptors are members of the GPCR superfamily and converge on shared signalling pathways involving cAMP generation, PKA activation, and CREB-mediated transcription [81], their combined activation amplifies these intracellular cascades, resulting in greater metabolic effects than either hormone alone. This coordinated enhanced postprandial endocrine signalling provides the physiological basis for dual GIP/GLP-1 therapies. Clinically relevant effects include augmented insulin secretion, improved hepatic insulin sensitivity, reduced appetite, and decreased caloric intake [81,86], within a broader pathophysiological framework that implicates GIP in lipid metabolism and insulin resistance [83].

These findings reshaped anti-obesity drug development by establishing a new benchmark with the first dual GLP-1/GIP receptor agonist, tirzepatide, demonstrating superior efficacy compared with single GLP-1 receptor agonists [87]. Overall, co-activation of GIP and GLP-1 receptors enhances insulin sensitivity, modulates lipid metabolism, and improves systemic energy regulation [81,86], effects exemplified by tirzepatide. In clinical studies, tirzepatide produced substantial body weight reductions (-15% with 5 mg, -19.5% with 10 mg, and -20.9% with 15 mg), achieved tight glycaemic control with reductions in HbA1c and high rates of reversion to normoglycaemia, and was associated with reduced progression to T2D and improvements in cardiometabolic risk factors, including triglycerides and blood pressure [88]. Although GIP is not currently regarded as an effective standalone therapy [30,89], accumulating evidence highlights its complex role in adipose tissue biology, including lipogenesis and fatty acid storage, suggesting future potential for targeted exploitation [81,86]. At present, however, its clinical relevance remains predominantly linked to co-activation with GLP-1 [81].

Interestingly, antagonism of GIPR has also been explored as a viable strategy for obesity and T2D management. The combined mechanism of GLP-1R agonism and GIPR antagonism is reported to augment glycaemic control and weight loss in preclinical experimental models [90]. Mechanistically, GLP-1R activation promotes negative energy balance *via* anorectic signalling and slowed gastric emptying, whereas GIPR antagonism is intended to counter pro-adipogenic GIP biology. Preclinical explorations further suggest co-ordinated engagement of these receptors may drive receptor internalisation and amplify intracellular signalling pathways relevant to body weight regulation and metabolic control [91]. From a molecular signalling perspective, the rationale for pursuing GIPR antagonism as a therapeutic strategy rests on a few paradoxical hypotheses. First, chronic stimulation of GIPR may itself induce functional antagonism. In primary adipocytes, prolonged exposure to an acylated GIPR agonist causes marked desensitisation to subsequent ligand stimulation, consistent with receptor uncoupling, altered intracellular trafficking and reduced downstream responsiveness [92]. According to this theory, GIPR internalisation is arrestin-dependent, and Gs-cAMP signalling is weakened in the absence of arrestins, indicating a signalling architecture distinct from GLP-1R and particularly susceptible to regulation by prolonged agonist exposure. Under this framework, both chronic agonism and direct antagonism could converge on reduced adipose GIPR tone, thereby limiting fatty-acid uptake, altering nutrient partitioning and reducing lipid sequestration in adipose tissue and liver [92]. The second hypothesis is CNS-centred; GIPR appears to signal through GABAergic neurones, whereas GLP-1R agonism engages glutamatergic circuits, suggesting that modulation of GIPR signalling may shift the inhibitory-excitatory balance within hypothalamic and brainstem feeding networks [93–95]. Consistent with this, neuronal and GABAergic GIPR deletion lowers body weight primarily through reduced food intake [93,94,96], and central GIPR neutralisation potentially enhances leptin receptor signalling [97]. The third hypothesis is that the superior efficacy of tirzepatide may not require a direct pro-weight-loss effect of GIPR agonism *per se* but instead may reflect biased agonism at GLP-1R, with relatively reduced β-arrestin recruitment and receptor internalisation compared with G-protein coupling, thereby sustaining anorectic GLP-1R signalling and enhancing weight reduction [98,99]. Collectively, these hypotheses provide a mechanistic basis for GIPR antagonism alone or in combination with GLP-1R agonism, where suppression of GIPR-dependent signalling may amplify anorectic and weight-lowering effects beyond those achieved with GLP-1 agonism alone [91,100–102]. At first glance, the clinical success of GIPR agonism and the emergence of GIPR antagonism appear contradictory. A concise reconciliation is that GIP biology is highly context-dependent. In chronic obesity and hyperglycaemia, native GIP signalling may be dysregulated, with receptor down-regulation/desensitisation contributing to impaired responsiveness; under those conditions, prolonged pharmacologic co-agonism with GLP-1 can shift the overall phenotype toward restored insulin secretion, appetite reduction, and weight loss. By contrast, antagonist strategies aim to blunt pro-adipogenic GIP tone or alter chronic receptor behaviour, especially when paired with GLP-1R agonism. Thus, the therapeutic outcome is shaped not only by whether GIPR is stimulated or blocked, but also by dose, duration, metabolic context, and co-engagement of companion pathways. At present, the major clinical evidence supports GIPR agonist-based co-agonism, whereas antagonist approaches remain promising but under investigation [81].

### GIP-based therapies in clinical use

#### Tirzepatide

*Tirzepatide*, approved in 2022, is a first-in-class SC dual incretin agonist that confers significant metabolic benefits across T2D, obesity, and metabolic liver conditions. It is a synthetically engineered 39-amino acid peptide that co-activates GIP and GLP-1 receptors. Its structural design incorporates non-natural amino acids, notably α-aminoisobutyric acid, to confer resistance to DPP-IV degradation. Conjugation of a C20 fatty di-acid moiety via a γ-glutamic acid linker and dual aminoethoxyethoxyacetyl spacers attached to a lysine residue enables high-affinity albumin binding and sustained systemic exposure. Collectively, these modifications extend its *t*_1/2_ to approximately five days, facilitating potent incretin activity with once-weekly dosing [22].

The clinical efficacy of tirzepatide has been rigorously established through the SURPASS Phase-3 programme in T2D, the SURMOUNT Phase-3 programme in obesity, and the Phase-2 SYNERGY-MASH trial targeting histological liver outcomes. In SURPASS-1 (NCT03954834), once-weekly tirzepatide (5–15 mg) achieved mean HbA1c reductions of 1.9–2.1%, accompanied by dose-dependent weight loss of 7–10 kg and high rates of normoglycemia over 40 weeks [103]. SURPASS-2 (NCT03987919) confirmed superiority over semaglutide 1 mg, with additional HbA1c reductions of 0.15%–0.45% and additional 2–5 kg weight loss [104]. In SURPASS-3 (NCT03882970), tirzepatide outperformed insulin degludec, reducing HbA1c by 1.9%–2.4% versus 1.3% and achieving weight reduction of 7.5–12.9 kg, contrasting with weight gain observed under insulin therapy [105]. SURPASS-4 (NCT03730662) enrolled patients with high CV risk and showed HbA1c reductions up to 2.6% and weight loss of 7–11 kg compared with insulin glargine, with fewer hypoglycaemic events [106]. SURPASS-5 (NCT04039503) corroborated these benefits in individuals on basal insulin, sustaining HbA1c reductions of 2.3–2.4% and weight loss approaching 10 kg [107].

The SURMOUNT clinical programme has established the profound and sustained weight-lowering efficacy of tirzepatide in obesity management. In the 72-week, randomised, placebo-controlled SURMOUNT-1 (NCT04184622) study, once-weekly therapy (5, 10, and 15 mg) resulted in mean body-weight reductions of 15.0%, 19.5%, and 20.9%, respectively, versus 3.1% with placebo [108]. SURMOUNT-2 (NCT04657003) confirmed similar efficacy in individuals with T2D, achieving weight reductions of 12.8% and 14.7% [109]. In SURMOUNT-3 (NCT04657016), tirzepatide administered after an intensive lifestyle intervention elicited an additional 18.4% weight loss, in contrast with a 2.5% weight regain with placebo, underscoring its synergy with healthy lifestyle modifications [110]. SURMOUNT-4 (NCT04660643) highlighted the chronic nature of obesity, revealing that treatment discontinuation led to about 14% weight regain, whereas continued therapy maintained and augmented prior weight reductions [88].

Beyond its effects on glycaemic regulation and weight management, tirzepatide has demonstrated clinically significant benefits in metabolic liver disease. In the SURPASS-3 study (NCT03882970), tirzepatide treatment reduced hepatic fat content by 40%–50% and improved visceral adiposity [111]. Complementing these imaging outcomes, the SYNERGY-NASH trial (NCT04166773) demonstrated histologic resolution of MASH without fibrosis progression in up to 62% of participants, with at least one-stage fibrosis improvement in 55% vs 30% with placebo [112].

### Emerging GIP agonism-based therapies

#### HS20094

*HS20094* (Jiangsu Hansoh Pharmaceutical, China, and Regeneron, U.S.A.) is a once-weekly SC dual GIP and GLP-1 agonist designed for balanced receptor engagement and optimised PK, enabling sustained weekly exposure. In first-in-human, single- and multiple-ascending-dose studies involving healthy participants (NCT05116410), HS20094 therapy exhibited a favourable PK profile, acceptable safety and preliminary evidence of metabolic efficacy. Subsequent evaluation in a Phase-2 randomised, double-blind, placebo- and active-controlled trial (NCT06118008) in adults with T2D (*n* = 54) employed a rapid dose-escalation regimen (5–20 mg) with semaglutide 1.0 mg and placebo as comparators. Short-term treatment demonstrated significant, dose-dependent reductions in HbA1c of 0.63%, 0.75%, and 0.84% at 5, 10, and 15 mg, respectively, coupled with a corresponding decrease in fasting plasma glucose of 2.87, 2.25, and 2.79 mmol/l and progressive, dose-related body weight loss of 1.27%, 2.51%, and 4.41% [113]. Long-term efficacy is currently being examined in a 32-week randomised Phase-2 trial (NCT06901648) in adults with inadequately controlled T2D, with tight glycaemic control as the primary endpoint. In parallel, HS20094 has advanced into Phase-2 and pivotal Phase-3 weight-management programmes in adults with overweight or obesity (NCT06118021, NCT06839664, NCT07156539), designed to assess sustained weight reduction and broader cardiometabolic benefits over 48 weeks. Comprehensive efficacy and safety outcomes from these ongoing studies remain pending for public disclosure.

#### HRS9531

*HRS9531* (Jiangsu Hengrui Pharmaceuticals and Kailera Therapeutics, China) is a once-weekly SC dual GLP-1 and GIP receptor agonist candidate. Its initial safety and tolerability were assessed in a Phase-1 randomised, placebo-controlled study (NCT05152277) comprising single (*n* = 60) and multiple (*n* = 30) ascending dose cohorts in healthy participants. A single 8.1 mg dose induced a 4.9% reduction in body weight by Day 8, whereas multiple dosing at 5.4 mg achieved approximately 10% weight loss by Day 36 [114]. In a subsequent Phase-2 study in adults with obesity without diabetes (NCT05881837; *n* = 249), HRS9531 produced dose-dependent weight loss of 5.4% (1.3 mg), 13.4% (3 mg), 14.0% (4.5 mg), and 16.8% (6 mg) compared with 0.1% with placebo at 24 weeks. Additionally, HRS9531 outperformed placebo in lowering blood pressure, improving glycaemic control, and reducing triglyceride levels. GI adverse events were mostly non-severe and transient [115]. A 52-week extension trial demonstrated maintenance of weight loss with weekly or biweekly dosing (NCT05881837), with up to a 17.97% mean reduction at week 32 and a preserved effect through week 52 [116]. Additional Phase-2 data from an independent obesity study (NCT06054698) showed that an 8 mg dose achieved a mean weight loss of 22.8% (placebo-adjusted 21.1%) at 36 weeks [117]. In parallel, a Phase-2 trial (NCT05966272) in adults with T2D (*n* = 199) incorporating multiple dose-escalation regimens demonstrated statistically significant, dose-dependent reductions in HbA1c by week-20 compared with placebo, accompanied by progressive body weight loss of up to 8.9% (6.9 kg). Additional benefits included reductions in fasting plasma glucose and triglycerides, along with improvements in hepatic transaminases (ALT and AST), underscoring its glycaemic and broader cardiometabolic effects [118].

#### VK2735

*VK2735* (Viking Therapeutics, U.S.A.) is a dual GLP-1 and GIP receptor agonist in clinical development as both once-weekly SC and once-daily oral formulations. The Phase-1 study (NCT05203237) with its SC formulation indicated a favourable efficacy, safety, and tolerability profile in healthy participants. In a subsequent Phase-2, 13-week, randomised, double-blind, placebo-controlled VENTURE trial (NCT06068946), treatment with VK2735 resulted in reductions in mean body weight from baseline, achieving losses of 9.1% and 14.7% in the 2.5 and 15.5 mg dose groups, respectively, compared with 1.7% with placebo [119]. The Phase-2 programme was expanded to include an oral tablet formulation, in which once-daily dosing of VK2735 in adults with obesity resulted in mean body-weight reductions of 2.3%, 7.0%, 8.7%, 11.1%, and 12.0% from baseline at doses of 15, 30, 60, 90, and 120 mg, respectively, compared with 1.3% with placebo [120].

#### CT-388

*CT-388* (Carmot Therapeutics Inc., U.S.A., and Roche, Switzerland) is a once-weekly, signal-biased dual GLP-1/GIP receptor agonist engineered to maintain potent cAMP signalling at both receptors while minimising β-arrestin recruitment, thereby attenuating receptor internalisation and supporting sustained metabolic efficacy. In a 4-week, ascending-dose Phase-1 (NCT04838405) study, CT-388 demonstrated a weight loss of 4%–8% compared with 0.5% with placebo, accompanied by improvements in fasting glucose and oral glucose tolerance in adults with overweight or obesity, irrespective of T2D status [121]. In a subsequent 24-week Phase-1b extension study (NCT06628362), CT-388 treatment resulted in a placebo-adjusted mean weight reduction of 18.8% and substantial glycaemic improvements among obese participants with dysregulated glycaemia. Adverse events were mainly GI and of low severity [122]. Ongoing Phase-2 trials (NCT06525935 and NCT06628362) are assessing the long-term (48-week) metabolic and hepatic efficacy of this dual incretin therapy.

#### SCHO-094

*SCHO-094* (SCOHIA Pharma and Huadong Medicine Co., Ltd., Japan) is a Phase-1 peptide co-agonist candidate targeting the GLP-1 and GIP receptors. In preclinical models, SCO-094 was reported to exhibit potent efficacy in reducing HbA1c by improving liver function, lowering triglycerides, and inhibiting hepatic fat accumulation. These preclinical findings support the clinical development of the agent for diabetes, obesity, and MASH. SCO-094 is currently being evaluated in a Phase-1 clinical study [123]. No further information has been publicly disclosed to date.

#### LY3537021

LY3537021 (Eli Lilly and Company, USA) is a once-weekly SC, long-acting (*t*_1/2_12 days) selective GIP receptor agonist. In a Phase-1 single- and multiple-ascending-dose study (NCT04586907; *n* = 85) conducted in healthy participants and adults with T2D (single-dose cohort, *n* = 47; multiple-dose cohort, *n* = 38), participants received single SC doses ranging from 0.3 to 25 mg or multiple once-weekly doses ranging from 4 to 25 mg. Primary endpoints of safety and tolerability were met, with infrequent GI adverse events. Secondary outcomes showed no delay in gastric emptying and dose-dependent weight loss persisting 35 days post-final dose. In participants with T2D, the 25-mg dose resulted in a mean weight loss of 3.14 kg vs 0.36 kg with placebo on Day 57, alongside transient fasting glucose reductions [124].

### Emerging GIP antagonism-based therapies

#### Maridebart cafraglutide/MariTide

*Maridebart cafraglutide/MariTide* (Amgen, U.S.A.) is a long-acting peptide-antibody conjugate comprising a fully human anti-GIPR antagonist monoclonal antibody conjugated to two GLP-1 analogue peptides. This molecular design confers prolonged systemic exposure, with a reported t_1/2_ of approximately 21 days, supporting once-monthly (Q4W) SC administration. In a Phase-1, randomised, double-blind study in adults with obesity without diabetes (NCT04478708, *n* = 110), single ascending doses (21–840 mg) and multiple Q4W doses (140, 280, 420 mg) were evaluated. By Day 85, mean body-weight reductions ranged from 7.2% (140 mg) to 14.5% (420 mg), with sustained weight loss persisting up to 150 days following the final dose. Treatment was tolerable with acceptable GI adverse events [125]. A subsequent Phase-2 dose-ranging, double-blind trial (NCT05669599) evaluated fixed Q4W doses (149, 280, and 429 mg) in individuals with obesity without diabetes (Cohort A; *n* = 465) and with T2D (Cohort B; *n* = 127). At 52 weeks, mean body weight reduction ranged from 12.3% to 16.2%; in cohort B, mean HbA1c reductions of approximately 1.2% to 1.6% were observed [126].

#### AT-7687

*AT-7687* (Antag Therapeutics, Denmark) is another preclinical GIPR antagonist candidate. When combined with liraglutide, it demonstrated around 16.3% weight reduction in cynomolgus monkeys, accompanied by a reduction in plasma glucose (30%), insulin (52%), triglycerides (39%), and cholesterol (48%) [127].

### Glucagon

Glucagon is a 29-amino-acid peptide primarily produced by pancreatic α cells and, to a lesser extent, by specific intestinal cells. Glucagon receptors (GCGRs), a member of the GPCR family, are predominantly expressed in hepatocytes and in the kidney, with substantial presence in the heart, pancreas, adipose tissue and the GI tract. Activation of GCGR increases intracellular cAMP and calcium levels, leading to PKA activation and subsequent modulation of multiple hepatic and systemic metabolic processes (Figure 2C) [128–130]. Within this physiological framework, glucagon serves as the principal counter-regulatory hormone to insulin in the maintenance of glycaemic homeostasis. During conditions of reduced substrate availability, particularly hypoglycaemia or prolonged fasting, glucagon stimulates hepatic gluconeogenesis and glycogenolysis while suppressing glycogenesis, thereby increasing circulating glucose concentrations. This hyperglycaemic nature of glucagon confers a therapeutic advantage for the clinical management of severe hypoglycaemia (glucose <54 mg/dl) induced by insulin therapy or prolonged fasting [131]. However, this same property of glucagon represents a limitation for the broader therapeutic application of selective GCGR agonism in T2D [128].

To date, multi-dimensional investigations into glucagon signalling have been actively pursued to advance innovative glucagon-based therapies for metabolic disorders. These efforts encompass GCGR agonism, antagonism, and polypeptide co-agonism strategies [132]. Accumulating evidence indicates that glucagon, conventionally viewed as a hyperglycaemic hormone, exerts metabolic effects that extend well beyond glucose regulation [133]. One of its key physiological actions is the stimulation of hepatic amino acid catabolism *via* GCGR activation, leading to increased expression of amino acid transporters and urea cycle enzymes. This enhances hepatic amino acid uptake and oxidation, resulting in systemic hypoaminoacidemia and underscoring a central role of glucagon in hepatic amino acid metabolism and the regulation of circulating amino acid levels [133].

Furthermore, experimental MASH studies demonstrate that in conjunction with GLP-1, GCGR agonism promotes fatty acid mobilisation from white adipose tissue, elevates adiponectin and fibroblast growth factor-21 levels, activates brown adipose tissue, and augments energy expenditure [134]. The same study showed that combined GLP-1R and GCGR activation ameliorated steatohepatitis and enhanced hepatic regeneration by modulating inflammation, oxidative stress, endoplasmic reticulum stress, mitochondrial dysfunction and alterations in carbohydrate metabolism. These preclinical findings highlight the hepatic benefits of glucagon signalling [134]. Furthermore, GCGR agonism suppresses adipose lipogenesis, stimulates lipolysis, and increases circulating concentrations of non-esterified fatty acids and ketone bodies. In preclinical models, GCGR activation enhances brown adipose tissue thermogenesis, whereas the associated increase in energy expenditure is more modest in humans [4,133]. Collectively, these observations position glucagon as an orchestrator of hepatic gluconeogenesis and glycogenolysis, amino acid metabolism, lipolysis and thermogenesis, thereby integrating metabolic flux across liver and adipose tissue compartments.

These properties provide a strong rationale for incorporating GCGR agonism into modern anti-obesity/MASH therapeutic strategies. Accordingly, GLP-1/GCGR co-agonists and GLP-1/GIP/GCGR tri-agonists are under development to synergise glucagon-driven energy expenditure and lipid metabolism with complementary incretin actions (Figure 2C) to achieve additive metabolic benefits [133].

### Emerging long-acting GCGR agonist

#### Efpegerglucagon

*Efpegerglucagon* (Hanmi Pharma, South Korea) is a once-weekly SC, long-acting GCGR agonist engineered using the LAPSCOVERY® platform, in which a glucagon analogue is covalently linked to a human immunoglobulin-G (IgG) Fc fragment via a non-peptidyl linker. Sustained GCGR activation augments hepatic glycogenolysis and gluconeogenesis, thereby counteracting recurrent hypoglycaemia in congenital hyperinsulinism [135]. In a randomised, single-ascending-dose Phase-1a study (NCT04032782) in healthy adults (*n* = 56), efpegerglucagon demonstrated acceptable PK characteristics, including slow absorption (*T*_max_ 46–68 h), extended *t*_1/2_ (77–101 h), and dose-dependent increases in fasting blood glucose of up to 1.5 mmol/l by Day 10 that persisted for approximately two weeks, supporting a once-weekly dosing regimen [136]. A subsequent 12-week multiple-ascending-dose Phase-1b trial (NCT04167553) in overweight or obese adults with dyslipidaemia and/or hypertension, with or without T2D, evaluated efpegerglucagon at 0.02, 0.04, and 0.06 mg/kg once weekly. Treatment showed dose-dependent increases in fasting plasma glucose, accompanied by modest body-weight reductions of 0.5%, 2.3%, and 2.6% in participants without diabetes and approximately 0.9% weight loss at 0.02 mg/kg in those with T2D. The 0.06 mg/kg dose was not tolerated in T2D due to hyperglycaemia; however, no serious treatment-emergent adverse events were reported [137]. Ongoing evaluation of efpegerglucagon in congenital hyperinsulinism is underway in the Phase-2a ACHIEVE trial (NCT04732416).

### Emerging GCGR-based therapies

#### Survodutide

*Survodutide* (Boehringer Ingelheim, Germany and Zealand Pharma, Denmark) is a long-acting, once-weekly SC, dual agonist of GLP-1 and GCGR, currently in Phase-3 evaluation for obesity and MASH. The peptide is engineered to retain full GLP-1R agonism while conferring partial GCGR activation. Structural modifications include C18 fatty acid conjugation to extend *t*_1/2_ (100 h), C-terminal amidation, and substitution at the second amino acid to resist DPP-IV-mediated degradation [138]. In Phase-1 studies involving healthy volunteers and participants with a BMI >27 kg/m^2^, survodutide achieved a maximum placebo-corrected weight loss of 13.8%, with dose-related GI adverse events [139]. In a Phase-2 trial (NCT04667377) in adults with obesity without diabetes (*n* = 387), 46 weeks of survodutide treatment resulted in dose-dependent weight loss of 6.2% (0.6 mg), 12.5% (2.4 mg), 13.2% (3.6 mg), and 14.9% (4.8 mg) vs -2.8% with placebo. Participants completing the 4.8 mg regimen achieved approximately 18.7% mean loss, with ≥20% loss in up to 40% of subjects; accompanied by a decrease in waist circumference and blood pressure [140]. A separate Phase-2 study (NCT04153929) in individuals with T2D (*n* = 413) demonstrated HbA1c reductions of 0.91% to 1.71% over 16 weeks, along with >5% body weight loss [141]. In a 48-week Phase-2 study (NCT04771273), 293 adults with biopsy-confirmed MASH (NAS activity score ≥4), F1–F3 fibrosis, MRI-PDFF liver fat fraction ≥8%, and liver stiffness >6.0 kPa underwent randomisation 1:1:1:1 to once-weekly SC survodutide (2.4 mg, *n* = 73; 4.8 mg, *n* = 73; 6.0 mg, *n* = 72) or placebo (*n* = 75) following 24-week dose escalation and 24-week maintenance. The prespecified primary endpoint including histologic MASH improvement, defined as a ≥2-point reduction in NAS with ≥1-point improvement in lobular inflammation or ballooning and no worsening of fibrosis was achieved in 47%, 62%, and 43% population from the 2.4-, 4.8-, and 6.0-mg survodutide groups, respectively, vs 14% with placebo (*P<*0.001 for the quadratic dose-response model). Prespecified secondary endpoints similarly favoured survodutide, including ≥30% reduction in liver fat by MRI-PDFF (63%, 67%, and 57% vs 14%) and ≥1-stage fibrosis improvement (34%, 36%, and 34% vs 22%). Prespecified secondary endpoints further supported efficacy with ≥30% liver fat reduction by MRI-PDFF occurring in 63%, 67%, and 57% vs 14%; ≥1-stage fibrosis improvement without MASH worsening was observed in 34%, 36%, and 34% vs 22% [142]. Mechanistically, this hepatic benefit is likely multifactorial, reflecting indirect effects of weight reduction and metabolic remodelling alongside putative direct glucagon receptor-mediated enhancement of hepatic lipid oxidation, although direct antifibrotic activity in humans remains unconfirmed. Survodutide is currently under Phase-3 evaluation across multiple programmes, including SYNCHRONIZE-1 (overweight/obesity without T2D; NCT06066515), SYNCHRONIZE-2 (overweight/obesity with T2D; NCT06066528), and SYNCHRONIZE-CVOT (CV outcomes; NCT06077864); LIVERAGE (MASH F2–F3; long-term outcomes; NCT06632444) and LIVERAGE-Cirrhosis (compensated MASH cirrhosis; NCT06632457).

#### Mazdutide

*Mazdutide* (Eli Lilly and Company, U.S.A. and Innovent Biologics, China) is a dual GLP-1 and glucagon receptor agonist administered once-weekly by SC injection. In a first-in-human, multiple ascending-dose Phase-1 trial (NCT04440345) conducted in adults with overweight or obesity (*n* = 60), mazdutide induced dose-dependent weight reduction ranging from 4.8% to 6.4% after 12 weeks of treatment, with low-grade GI adverse events [143]. In a complementary randomised, placebo-controlled Phase-1b study (NCT04466904) in individuals with T2D (*n* = 43), mazdutide resulted in clinically meaningful reductions in HbA1c, fasting and post-prandial glucose, with a safety and tolerability profile comparable to dulaglutide and placebo [144]. A subsequent 20-week Phase-2 trial in Chinese adults with T2D demonstrated HbA1c reduction of 1.41% to 1.67% and body-weight loss up to 7.1% vs placebo [145]. Among participants with overweight or obesity without diabetes (*n* = 248), a 24-week randomised, double-blind, placebo-controlled Phase-2 study (NCT04904913) revealed dose-dependent body-weight reductions of 6.7% (3 mg), 10.4% (4.5 mg), and 11.3% (6 mg) [146]. These findings were subsequently corroborated in the Phase-3 GLORY-1 trial (NCT05607680), which enrolled adults with overweight or obesity (*n* = 610). At 32 weeks, mean placebo-adjusted body-weight changes were 10.1% for 4 mg and 12.6% for 6 mg mazdutide, compared with a 0.5% gain in the placebo group. Clinically meaningful weight loss (≥5%) occurred in 73.9% and 82.0% of participants receiving 4.5 and 6 mg, respectively, accompanied by improvements in cardiometabolic parameters and a tolerability profile dominated by limited clinical severity GI adverse events [147].

#### Pemvidutide

*Pemvidutide* (Altimmune, Inc., U.S.A.) is a once-weekly SC peptide therapy under investigation for the management of obesity and metabolic liver disease. It functions as a balanced (1:1) dual agonist of GLP-1R and GCGR, mechanistically integrating GLP-1-mediated appetite suppression and weight reduction with GCGR-driven hepatic lipid mobilisation and augmented energy expenditure [148]. In a Phase-1 trial (NCT04561245) in adults with overweight or obesity (*n* = 100), pemvidutide treatment over 12 weeks exhibited favourable PK characteristics, along with a well-tolerated safety profile. This was followed by a 12-week, Phase-1b study (NCT05006885) in overweight or obese participants with MASLD (*n* = 94), in which pemvidutide resulted in a substantial reduction in hepatic fat content (68.5%). The maximal effects were observed in the 1.8 mg cohort, which also exhibited reductions in body weight (4.3%) and plasma ALT concentrations (13.8 IU/L), with no severe adverse events [149]. A subsequent 12-week extension study (NCT05292911) in adults with MASLD (*n* = 64; 26.6% with T2D) showed sustained hepatic benefits after 24 weeks of pemvidutide treatment. The primary endpoints of relative liver fat reduction by MRI-PDFF were met, with mean reductions of 56.3% (1.2 mg), 75.2% (1.8 mg), and 76.4% (2.4 mg) compared with 14.0% for placebo. Key secondary endpoints were also concordant, showing absolute liver fat reductions of 11.2%, 17%, and 15.6% vs 1.6%, ALT level reductions of 13.3 IU/l, 13.7 IU/l, and 15.2 IU/l vs 2.2 IU/l, and body weight reduction of up to 6.2%, supporting durable antisteatotic and metabolic efficacy [150]. Furthermore, in the Phase-2b IMPACT trial (NCT05989711), 212 adults with biopsy-confirmed F2–F3 MASH, with or without T2D, underwent randomisation 1:2:2 to once-weekly pemvidutide (1.2 mg, *n* = 53; 1.8 mg, *n* = 106) or placebo (*n* = 53). At week 24, the dual primary hepatic endpoints showed MASH resolution without fibrosis worsening in 58% and 52% of the 1.2 and 1.8 mg groups, respectively, compared with 20% with placebo, whereas at least one-stage fibrosis improvement without worsening of MASH was not significantly different at 33% and 36% vs 28%. Secondary endpoints were nevertheless favourable, including concurrent MASH resolution with fibrosis improvement in 24% vs 14%, relative MRI-PDFF liver fat reductions of 52.0% and 57.7% compared with 11.4%, liver fat normalisation in 31% and 44% vs 4%, and ALT level declines of 34.6 IU/l and 34.4 IU/l vs 10.3 IU/l, collectively indicating substantial anti-steatotic and hepatocellular efficacy despite the absence of a significant categorical fibrosis response at this interim analysis [151]. In adults with overweight or obesity without diabetes, 48-week findings from the Phase-2 MOMENTUM trial (NCT05295875; *n* = 391) revealed robust body-weight reductions of 10.3%, 11.2%, and 15.6% with pemvidutide 1.2, 1.8, and 2.4 mg regimens, respectively, compared with 2.2% for the placebo group. Concomitant cardiometabolic benefits were evident with systolic blood pressure reductions of 2.3, 1.6, and 4.6 mmHg and diastolic blood pressure reductions of 2.1, 1.0, and 2.9 mmHg, whereas placebo was associated with increases of 3.5 and 1.8 mmHg, respectively. Mean heart rate changes were modest at 0.1, 3.1, and 2.5 beats per minute vs −1.4 beats per minute with placebo. These effects were accompanied by reductions in waist circumference and serum lipids [152]. Currently, a Phase-2 RECLAIM trial (NCT06987513) is assessing the efficacy and safety of once-weekly pemvidutide 2.4 mg in 100 adults with alcohol use disorder.

#### Efinopegdutide

*Efinopegdutide* (Hanmi Pharma, South Korea and Merck MSD, U.S.A.) is a once-weekly SC agonist of GLP-1 and GCG receptors. This co-agonist consists of a chemically synthesised GLP-1/GCG hybrid peptide covalently conjugated to a human IgG-Fc fragment *via* a flexible polyethylene glycol (PEG) linker, conferring prolonged PK stability and sustained receptor engagement. In a Phase-1 trial on healthy volunteers (*n* = 40), efinopegdutide demonstrated an acceptable safety profile and predictable PK profile following single and multiple ascending dose administrations [153]. In a subsequent 26-week, Phase-2 study involving adults with obesity (NCT03486392; *n* = 474), efinopegdutide treatment resulted in mean body weight reductions of 6.8% (5 mg), 8.1% (7.4 mg), and 10.0% (10 mg), respectively. This effect was accompanied by higher incidences of nausea and vomiting, relative to placebo [154]. In a parallel 12-week Phase-2 study (NCT03586830) involving individuals with T2D and obesity (*n* = 195), efinopegdutide achieved placebo-subtracted weight reductions of 4.6%, 5.9%, and 7.2% with 5, 7.4, and 10 mg doses, respectively, without significant HbA1c improvement [155]. In a 24-week Phase-2a trial (NCT04944992), enrolling adults with hepatic steatosis (≥10% liver fat by MRI-PDFF; BMI 25–50 kg/m^2^; approximately 33% with T2D; *n* = 145), once-weekly 10 mg efinopegdutide treatment achieved a mean relative liver fat reduction of 72.7% compared with 42.3% for semaglutide 1 mg. The change in body weight was comparable between groups (8.5% vs 7.1%). GI adverse events were more frequent with efinopegdutide, leading to modest treatment discontinuation [156]. The greater hepatic response observed with efinopegdutide, relative to semaglutide, may be attributable to additional GCGR-mediated direct hepatic effects, consistent with the more pronounced reduction in liver fat despite comparable weight loss.

#### Cotadutide

*Cotadutide* (MedImmune LLC/ AstraZeneca, United Kingdom) is a rationally engineered dual agonist peptide activating GCGR and GLP-1R. Structurally, it is related to the oxyntomodulin peptide with incorporated amino acid substitutions alongside chemical modifications to optimise receptor selectivity, enhance metabolic stability, and improve PK performance. The peptide comprises 39-amino acid residues, conferring a molecular architecture tailored for balanced activation of both GLP-1 and glucagon signalling [157]. Its clinical evaluation in multiple Phase-2 trials has demonstrated its metabolic and renal efficacy across diverse populations. In a Phase-2a randomised, placebo-controlled study (NCT03244800), once-daily cotadutide at doses of 50-300 μg resulted in a 21.5% reduction in postprandial glucose excursions and a 3.4% reduction in body weight [158]. In the subsequent Phase-2b trial (NCT04515849) involving 248 adults with T2D and chronic kidney disease, once-daily SC administration of cotadutide at 100, 300, and 600 μg doses produced dose-related reductions in urinary albumin-to-creatinine ratio (39.9% and 46.0% for 300 and 600 μg, respectively) and an improvement in estimated glomerular filtration rate (+5.5 ml/min/1.73 m^2^ at 600 μg) at 26 weeks. These benefits were accompanied by reductions in body weight (up to 6.5% vs 2.1% with placebo) and HbA1c (0.9% vs 0.3% with placebo) [159]. The 19-week Phase-2 PROXYMO trial established clinical proof-of-concept for cotadutide as a protective therapy in MASH. In this randomised study (NCT04019561), 74 adults with biopsy-confirmed non-cirrhotic MASH and fibrosis were enrolled (BMI ≥30 kg/m^2^, NAS ≥4, fibrosis stage F1–F3, MRI-PDFF liver fat ≥10%), with inclusion permitted irrespective of T2D status; the enrolled cohort was predominantly stage F2–F3 fibrosis (76%), and 55% had T2D. Once-daily cotadutide administered at 300 or 600 μg produced positive hepatic and metabolic effects, with the 600 μg dose yielding significant improvements vs placebo in absolute hepatic fat fraction (least-squares mean difference, -5.0% [95% CI, -8.5 to -1.5]), ALT (-23.5 U/l [-47.1 to -1.8]), AST (-16.8 U/l [-33.0 to -0.8]), and body weight (-2.34 kg [-4.71 to -0.002]), whereas the 300 μg dose elicited more modest, largely non-significant effects. Treatment-emergent GI adverse events were more frequent with cotadutide than with placebo and resulted in discontinuation rates of 16.7% (600 μg) and 7.7% (300 μg) compared with 4.2% placebo group [160]. Collectively, cotadutide demonstrates a compelling therapeutic profile for integrated management of metabolic and renal complications in T2D, with ongoing investigations evaluating its potential in obesity and MASH.

### Emerging GLP-1, GIP, and GCGR tri-agonist therapies

Triple agonists targeting GLP-1, GIP, and glucagon receptors represent a next-generation advancement in the emerging therapeutic landscape for metabolic diseases.

#### Retatrutide

*Retatrutide* (Eli Lilly and Company, U.S.A.) is a once-weekly SC therapy. This 39-amino acid single-peptide tri-agonist is engineered for balanced and sustained activation of GLP-1, GIP, and GCG receptors to enhance metabolic efficacy. Its design features a stabilised peptide backbone conjugated to a fatty diacid moiety, conferring extended PK characteristics (*t*_1/2_ around 6 days) compatible with weekly administration [161]. In a Phase-1b proof-of-concept study (NCT04143802) in individuals with T2D (*n* = 72), multiple once-weekly dose cohorts (0.5, 1.5, 3, and 3/6 mg, and escalation regimens of 3, 6, 9, and 12 mg) demonstrated dose-dependent improvements in glycaemic control and body weight. In higher-dose groups, HbA1c reductions of approximately 1.2% to 1.6% were observed, with placebo-adjusted weight loss reaching 8.96 kg in the 3, 6, 9, and 12 mg escalation cohorts and mild-to-moderate GI adverse events [162]. In a 48-week Phase-2 obesity trial (NCT04881760; *n* = 338), once-weekly retatrutide at 1 mg, 4 mg (starting dose 2 or 4 mg), 8 mg (starting dose 2 or 4 mg), or 12 mg (starting dose 2 mg) achieved sustained weight reduction, with least-squares mean weight change of 17.5% at week 24 and 24.2% at week 48 with the 12 mg dose group. GI tolerability was dose-dependent and consistent with incretin-based therapies [163]. A pre-specified MASLD imaging sub-study from this trial (*n* = 98) demonstrated marked, dose-related reductions in hepatic steatosis at week 24 (42.9% with 1 mg, 57.0% with 4 mg, 81.4% with 8 mg, and 82.4% with 12 mg retatrutide). In the 12 mg cohort, high rates of liver-fat normalisation (<5%) were observed at week 24 (86%) and week 48 (93%). Biomarker changes were directionally consistent with improved insulin sensitivity and lipid metabolism, although liver enzymes and fibrosis scores did not show consistent placebo-adjusted changes over 48 weeks [164]. Furthermore, a Phase-2 study (NCT04867785) involving adults with T2D (*n* = 281), once-weekly retatrutide (12 mg escalation) produced up to 2.02% reduction in HbA1c at week 24 compared with 0.01% with placebo and 1.41% with dulaglutide (1.5 mg). In the same cohort, body-weight reduction of 16.94% was observed at 36 weeks. No episodes of severe hypoglycaemia were reported [165]. Retatrutide is currently undergoing Phase-3 clinical evaluation across multiple metabolic and weight-related comorbidities, including TRANSCEND-T2D-2 (NCT06260722) and the TRIUMPH programme, encompassing obesity or overweight with knee osteoarthritis (TRIUMPH-4; NCT05931367), maintenance of weight loss (TRIUMPH-6; NCT06859268), obesity with chronic low back pain (TRIUMPH-7; NCT07035093), and obesity or overweight without T2D (TRIUMPH-8; NCT07232719).

#### Efocipegtrutide

*Efocipegtrutide* (Hanmi Pharma, South Korea) is a once-weekly, long-acting peptide developed using the LAPS® (Long-Acting Protein/Peptide System) platform, which transiently conjugates a modified peptide to an Fc fragment to impart albumin-like PK. This approach enables sustained systemic exposure and once-weekly SC dosing while preserving balanced receptor engagement [166]. Early clinical development has characterised the safety, PK, and preliminary efficacy of efocipegtrutide in Phase-1 single-ascending-dose studies (NCT03374241) in healthy obese participants (*n* = 41) and multiple-ascending-dose studies (NCT03744182) in obese individuals with MASLD (*n* = 66) [167]. In the latter study, 12 weeks of once-weekly efocipegtrutide reduced hepatic steatosis, with mean relative liver fat declining at week 12 ranging from 19.6% to 81.2% across dose cohorts vs 5.7% with placebo. In the highest-dose cohort, all evaluable participants (8/8) met ≥50% relative liver fat reduction thresholds at week 12 [167]. These hepatic benefits are most plausibly attributable to both indirect metabolic improvements, through enhanced glycaemic control and weight loss, and putative direct GCGR-mediated hepatic signalling. A Phase-2b trial in patients with biopsy-confirmed MASH (NCT04505436) is currently underway in the U.S.A. and South Korea [168].

### Amylin

**Figure 3:**
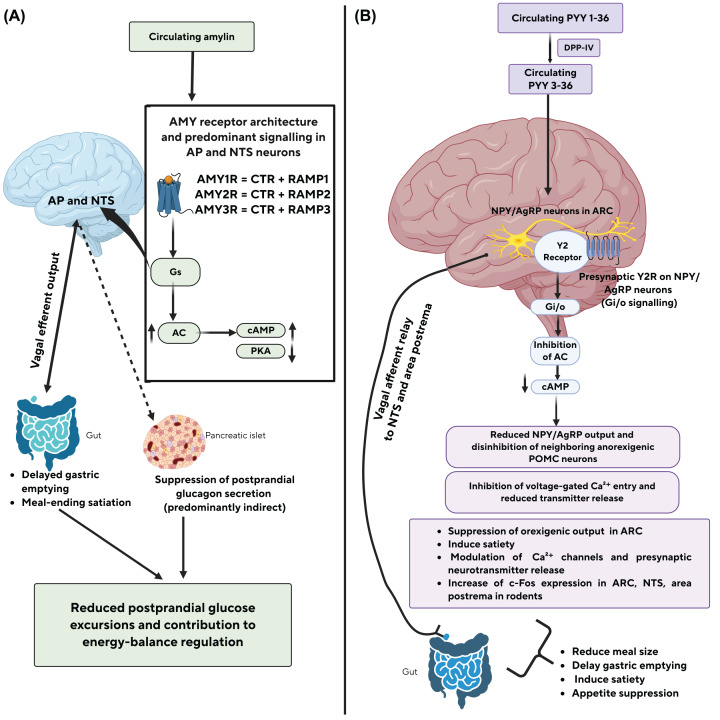
Mechanisms of Amylin and PYY Action in Gut–Brain Circuits Controlling Appetite and Postprandial Metabolism Mechanistic overview of (**A**) amylin and (**B**) PYY signalling in gut–brain circuits regulating appetite and postprandial metabolism. Figure created with BioRender.com Abbreviations: AC, adenylyl cyclase; AgRP, agouti-related peptide; AMYR, amylin receptor; AP, area postrema; ARC, arcuate nucleus; cAMP, cyclic adenosine monophosphate; Ca^2+^, calcium ion; CTR, calcitonin receptor; DPP-IV, dipeptidyl peptidase-IV; Gi/o, inhibitory guanine nucleotide-binding protein i/o; Gs, stimulatory guanine nucleotide-binding protein s; NPY, neuropeptide Y; NTS, nucleus tractus solitarius; PKA, protein kinase A; POMC, pro-opiomelanocortin; PYY, peptide YY; RAMP, receptor activity-modifying protein; Y2R, Y2 receptor.

Amylin, also known as islet amyloid polypeptide, is a 37-amino acid peptide co-secreted with insulin by pancreatic β-cells in response to nutrient intake and is an important regulator of glycemia, satiety, and body weight homeostasis [169–171]. In T1D, loss of β-cell function results in hypoamylinaemia, whereas in T2D, amylin secretion is often initially increased but declines with progressive β-cell dysfunction, leading to relative amylin deficiency [169,170,172,173]. Given its central role in gut-brain signalling and metabolic control, distributed circulating amylin concentrations in diabetes are likely to contribute to dysregulation of appetite and weight homeostasis [169,170]. Physiologically, amylin reduces food intake, delays gastric emptying, suppresses postprandial glucagon secretion, and acts as a long-term adiposity signal, thereby contributing to overall energy balance [169–171,174]. These actions are mediated by amylin receptors (AMYRs), heterodimeric class-B GPCRs composed of calcitonin receptor (CTR) and one of three receptor activity-modifying proteins (RAMP1, RAMP2, or RAMP3). These generate distinct receptor subtypes: AMY1 (CTR + RAMP1), AMY2 (CTR + RAMP2), and AMY3 (CTR + RAMP3) [175,176]. AMYRs are expressed predominantly in the area postrema (AP) and NTS within the hindbrain. Upon ligand binding, AMYRs signal predominantly through Gs-dependent activation of adenylyl cyclase, leading to increased cyclic AMP and downstream protein kinase A activity, with additional engagement of the ERK1/2 pathway (Figure 3A). In the AP/NTS, these signalling events mediate acute anorectic responses, including meal termination and reduced energy intake, whereas hypothalamic AMYR signalling may contribute to long-term energy homeostasis and enhanced leptin responsiveness. Peripheral metabolic effects are mediated largely through central autonomic outputs and include slowed gastric emptying, suppression of glucagon secretion, and reduced hepatic glucose production, collectively improving postprandial glycaemic control [177–179]. The advancing understanding of amylin pharmacology has spurred the development of synthetic amylin analogues and amylin receptor agonists as potential therapies for metabolic conditions.

### Amylin analogue in clinical use

#### Pramlintide

*Pramlintide* (AstraZeneca, U.K.) is a 37-amino acid synthetic analogue of human amylin, engineered with three proline substitutions (A25P, S28P, and S29P) to preclude amyloid fibril formation while preserving full amylino-mimetic activity. It suppresses post-prandial glucagon secretion, slows gastric emptying, and promotes satiety. Approved by the FDA in 2005, pramlintide is indicated as an adjunct to prandial insulin therapy, administered subcutaneously before major meals in adults with T1D or insulin-treated T2D who exhibit suboptimal glycaemic control on insulin alone [180,181]. Beyond diabetes management, pramlintide has demonstrated anti-obesity efficacy. In obese non-diabetic adults, a 16-week randomised study and a 12-month multicentre study showed sustained, dose-dependent body-weight reductions of 6%–8% with 120–360 μg twice or thrice-daily regimens, with enhanced effects when combined with structured lifestyle intervention [182].

### Emerging amylin-based therapies

#### Cagrilintide

*Cagrilintide* (Novo Nordisk, Denmark) is a once-weekly SC amylin analogue developed to recapitulate endogenous amylin pharmacology with extended systemic exposure for sustained anorexigenic signalling. Mechanistically, it binds and activates AMY1/AMY2/AMY3 (CTR-RAMP complexes) and the calcitonin receptor with an amylin-specific binding profile that preferentially suppresses appetite and delays gastric emptying [183]. In a randomised, placebo-controlled, multiple-ascending-dose Phase-1b trial (NCT03600480) in adults with overweight or obesity (*n* = 95), once-weekly cagrilintide (0.16–4.5 mg) co-administered with semaglutide 2.4 mg demonstrated a safety profile primarily characterised by non-severe GI adverse events. At week 20, cagrilintide produced clear exploratory weight-loss signals, yielding mean body-weight reductions of 15.7% with a 1.2 mg dose and 17.1% with a 2.4 mg dose [184]. A subsequent, Phase-2 dose-finding trial (NCT03856047) in adults with overweight or obesity without diabetes demonstrated dose-dependent body-weight reductions of 6.0%–10.8% compared with 3.0% with placebo. Notably, the 4.5 mg dose achieved superior efficacy to liraglutide 3.0 mg (10.8% vs 9.0%) [185]. The Phase-3 REDEFINE 1 trial (NCT05567796) evaluating cagrilintide 2.4 mg and semaglutide co-administration in adults with overweight or obesity (*n* = 302) reported a mean body weight reduction of 11.5% at week 68 [186].

#### CagriSema

*CagriSema* (Novo Nordisk, Denmark) is a once weekly, fixed-dose SC co-formulation of cagrilintide (amylin analogue) and semaglutide (GLP-1 analogue), delivered *via* a dual-chamber pen with dose escalation to 2.4 mg of each component. This combination engages complementary satiety and glucoregulatory pathways through concurrent amylin (CTR-RAMP complexes) and GLP-1 receptor activation, potentiating reductions in energy intake and enhancing glycaemic control. A Phase-1b trial (NCT03600480) established the feasibility of once-weekly cagrilintide (0.16–4.5 mg) co-administered with semaglutide 2.4 mg for weight management [184]. In a Phase-2 trial (NCT04982575) in adults with T2D on metformin ± SGLT2 inhibitor (*n* = 92), CagriSema achieved superior reductions in HbA1c (2.2% against 1.8 with semaglutide and 0.9% with cagrilintide), body-weight (15.6% against 5.1% and −8.1%, respectively), and fasting plasma glucose (3.3 mmol/l against 2.5 and 1.7 mmol/l, respectively) at week 32 [187]. The Phase-3a REDEFINE 1 study (NCT05567796) in adults with overweight or obesity without diabetes (*n* = 3417) revealed a week 68 mean body-weight reduction of 20.4% with CagriSema, accompanied by frequent but largely transient GI adverse events [186]. In the REDEFINE 2 trial (NCT05394519), T2D adults with overweight or obesity (*n* = 1206) exhibited greater body weight (13.7%) and HbA1c (1.8%) reductions following 68 weeks of treatment with CagriSema than with placebo [188].

#### Petrelintide

*Petrelintide* (Zealand Pharma, Denmark and Roche, Switzerland) is a once-weekly SC therapy. This 36-amino acid acylated amylin analogue is engineered for high chemical and physical stability, including minimal fibrillation at neutral pH and potent activation of amylin and calcitonin receptors. It promotes satiety, reduces energy intake, and exhibits glucometabolic effects, including glucagon suppression, with PK supporting durable weight-loss therapy [189]. In a first-in-human, randomised, double-blind, placebo-controlled single-ascending-dose Phase-1 trial (NCT05096598) in healthy lean or overweight men (*n* = 56), petrelintide demonstrated favourable safety and a mean *t*_1/2_ of approximately 10 days, consistent with once-weekly dosing. Single doses (0.7, 1.4, and 2.4 mg) produced dose-dependent glucagon suppression and early weight-loss signals, with mean 7-day body-weight reductions of 2.6%, 3.6%, and 4.2%, respectively. A subsequent multiple-ascending-dose Phase-1 trial (NCT05613387) in healthy lean or overweight adults confirmed these effects. In part 1 (*n* = 20), petrelintide treatment yielded 5.3% (0.6 mg) and 5.1% (1.2 mg) mean body-weight reductions at week 6 compared with 0.4% with placebo. The 16-week extension (part 2; *n* = 48) achieved up to 8.6% weight loss with higher doses compared with 1.7% with placebo [190]. Petrelintide is currently in Phase-2b development. The ongoing program comprises ZUPREME-1 (NCT06662539), a 42-week trial in adults with overweight or obesity without T2D, and ZUPREME-2 (NCT06926842) in adults with overweight or obesity with T2D. These studies will determine the long-term safety, tolerability, and metabolic efficacy of petrelintide.

#### Amycretin

*Amycretin* (Novo Nordisk, Denmark) is a unimolecular dual agonist designed to concurrently activate GLP-1 and amylin receptors, thereby integrating incretin-mediated glycaemic regulation with amylin-driven satiety and energy intake suppression. The peptide incorporates C18 diacid-based acylation, enabling reversible albumin binding and prolonged systemic exposure, an N-terminal 2-aminoisobutyric acid substitution to reduce DPP-IV susceptibility, and C-terminal amidation to enhance stability. Its oral preparation is co-formulated with SNAC to facilitate GI absorption and enable once-daily tablet administration, whereas a peptide formulation supports once-weekly SC injection [191]. In a randomised, double-blind, placebo-controlled Phase-1b/2a dose-ranging trial (NCT06064006) in adults with overweight or obesity without diabetes (*n* = 125), amycretin injection produced robust, dose-dependent body-weight reductions of 9.7% (1.25 mg, week 20), 16.2% (5 mg, week 28), 22.0% (20 mg, week 36), and 24.3% (60 mg, week 36), with tolerability limited predominantly by transient GI adverse events [192]. In a Phase-1 multiple-ascending-dose trial (NCT05369390) in adults with overweight or obesity without diabetes (*n* = 144), oral amycretin treatment produced dose-dependent body-weight reductions of 10.4% (50 mg once-daily) and 13.1% (50 mg twice-daily), accompanied by reductions in fasting plasma glucose [193].

#### Eloralintide

*Eloralintide* (Eli Lilly and Company, U.S.A.) is a once-weekly SC, selective amylin receptor agonist in development for obesity and related cardiometabolic complications. This 37-amino-acid fatty-acylated amylin analogue features three non-coded amino acid substitutions, a methylene thioacetal bridge replacing the native disulfide bond, and a C20 fatty diacid conjugate at Lys26. These modifications enhance chemical stability, reduce immunogenicity risk, confer preferential AMY1 activity over AMY3, and extend exposure to enable weekly dosing [194]. In a Phase-1 single-ascending-dose study (NCT05295940; *n* = 48), healthy adults received single SC eloralintide doses of 0.04–12 mg. The primary endpoint of safety and tolerability was met at week 4 with a mean per cent change from baseline in body weight of -2.5% and -4.4%, respectively, vs placebo (+0.6%) [194]. In a 48-week Phase-2 placebo-controlled trial (NCT06230523; *n* = 263) in adults with obesity (BMI ≥30 kg/m^2^) or overweight (BMI ≥27 kg/m^2^) plus ≥1 weight-related comorbidity (without T2D), once-weekly SC eloralintide (1, 3, 6, or 9 mg, or escalation regimens 6/9 mg and 3/6/9 mg) met the primary endpoint of superior percentage body-weight reduction at week 48 (-9.5%, -12.4%, -17.6%, -20.1%, -19.9%, and -16.4%, respectively, vs -0.4% with placebo). Secondary endpoints, including absolute weight change and BMI reduction, also improved, with mean weight loss up to 21 kg and BMI reduction up to 7.9 kg/m^2^. Treatment discontinuations due to GI adverse events were ≤12% in most groups (21% in the 6 mg arm). Adverse events were mainly fatigue-related, with nausea in 11%, 13%, 64%, 33%, 54%, and 25% of active arms vs 14% with placebo [195].

#### AZD6234

*AZD6234* (AstraZeneca, U.K.) is a long-acting, peptide-based selective amylin receptor agonist designed to preferentially engage amylin receptor signalling while minimising off-target calcitonin receptor activation, with structural optimisation supporting once-weekly SC administration [196]. The first-in-human Phase-1 single-ascending-dose study (NCT05511025) in adults with overweight or obesity evaluated safety, tolerability, and PK following single SC doses of AZD6234.

### Peptide YY

PYY is a 36-amino acid gut peptide hormone co-secreted with GLP-1 and oxyntomodulin by enteroendocrine L-cells in the ileum and colon in response to nutrient ingestion [197–199]. In humans, PYY circulates in two bioactive isoforms, PYY_1–36_ and PYY_3–36_, with PYY_3-36_ constituting the predominant circulating form generated through post-secretory N-terminal cleavage of PYY_1–36_ by DPP-IV enzyme [197]. This enzymatic truncation removes the N-terminal dipeptide to produce PYY_3–36_, the principal biologically active circulating isoform. Subsequent C-terminal trimming yields shorter, inactive fragments such as PYY_3–34_ [198]. Postprandial PYY levels rise proportionally to caloric intake, with preferential stimulation by fat- and protein-rich meals [197,200]. Circulating PYY_3–36_ suppresses food intake *via* presynaptic Y2 receptors on arcuate NPY/AgRP neurons and via Y2R-expressing vagal afferents projecting to the NTS/area postrema. Y2R signalling reduces adenylyl cyclase/cAMP and presynaptic Ca^+2^-dependent neurotransmitter release, decreasing NPY/AgRP output and disinhibiting POMC neurons (Figure 3B). Net effects include reduced meal size, increased satiety, and delayed gastric emptying. Human imaging supports modulation of corticolimbic responses to food cues, while preclinical studies implicate additional mesolimbic circuitry [198,201–204]. This signalling pathway in animal models is suggested to reduce food intake and body weight and improve insulin sensitivity [205,206]. In addition to its central effects, PYY exerts peripheral actions by delaying gastric emptying and intestinal transit, thereby contributing to the colonic brake and prolonging postprandial satiety [197,207]. PYY_3–36_ is believed to increase insulin sensitivity independent of weight loss [205,208–210]. PYY signalling operates within an integrated network of gut-derived hormones that communicate bi-directionally with the CNS via the gut–brain axis to regulate appetite, energy intake, and body weight [211]. Metabolic surgeries further substantiate this framework by demonstrating sustained weight loss partly through enhanced postprandial secretion of GLP-1 and other satiety-promoting gut hormones, including GIP, PYY, and oxyntomodulin, thereby remodelling gut-brain signalling and promoting appetite suppression. This highlights the pharmacological relevance of PYY signalling as a target for achieving sustained weight loss [30]. In this context, endogenous elevation of GLP-1, GIP, and PYY concentrations may be achieved by targeting multiple GPCRs expressed on intestinal enteroendocrine cells, including GPR119, GPR40, and TGR5 [30].

### Emerging PYY-based therapies

#### NNC0165-1875

*NNC0165-1875* (Novo Nordisk, Denmark) is a long-acting PYY_3–36_ analogue optimised through sequence variant screening to preserve Y2 receptor selectivity and stability, with fatty di-acid derivatisation conferring a PK profile suitable for once-weekly SC administration and sustained anorexigenic signalling [205]. In a Phase-1 study (NCT03707990) on adults with overweight or obesity (*n* = 88), single SC doses (0.1–2.1 mg; part 1, *n* = 40) as monotherapy or combined with semaglutide 0.25 mg demonstrated acceptable tolerability, with mild and manageable GI adverse events and occasional mild injection-site reactions. No anti-drug antibodies were detected, and the PK-PD profiles supported once-weekly administration [198]. In a subsequent Phase-2 study in adults with obesity (NCT04969939), participants underwent a 16-week, single-site dose-escalation phase (part 1), followed by a multicentre, open-label 32-week semaglutide 2.4 mg run-in (part 2). Eighty-three participants, achieving a mean 14.3% weight reduction during the run-in, were randomised to receive add-on NNC0165-1875 (1.0 mg, *n* = 47; 2.0 mg, *n* = 8) or placebo (*n* = 28) for 16 weeks. Add-on NNC0165-1875 1.0 mg produced a modest incremental weight reduction relative to semaglutide alone, while HbA1c and fasting plasma glucose remained stable, consistent with the predominantly non-diabetic cohort. GI adverse events limited tolerability, contributing to frequent discontinuations in part 1 and precluding escalation to the 2.0 mg regimen [198].

#### NNC0165-1562

*NNC0165-1562* (Novo Nordisk, Denmark) is a long-acting PYY analogue under clinical investigation for obesity management, with limited publicly available data. A first-in-human Phase-1 study (NCT02568306) conducted in adults with overweight or obesity (*n* = 93) evaluated single and multiple SC doses of NNC0165-1562 against placebo, focusing primarily on safety, tolerability, and PK aspects. In a separate Phase-1 randomised trial (NCT03574584), ascending multiple weekly doses of NNC0165-1562 were co-administered with semaglutide over 20 weeks to assess treatment-emergent adverse events and steady-state PK profiles of both agents. This investigation aimed to establish tolerability and dose feasibility without predefined weight loss efficacy endpoints.

#### Y14 peptide

*Y14 peptide* (Imperial College London, U.K.) is an extended-release PYY analogue derived from the PYY_1-36_ scaffold by N-terminal residue deletion and six strategic amino acid substitutions, thereby preserving the receptor-binding C-terminal pharmacophore. Composed solely of natural L-amino acids without covalent derivatisation, Y14 is formulated with zinc chloride to establish a sustained-release SC depot, facilitating administration at 7- to 14-day intervals. These modifications confer selective agonism at neuropeptide Y2 receptors, thereby potentiating anorexigenic signalling to suppress appetite and curtail energy intake in obesity management [212]. In a randomised, placebo-controlled, first-in-human Phase-1 trial (NCT03673111) in overweight or obese adults, Y14 was assessed for safety, tolerability, and PK characteristics. Part A was a blinded single-ascending-dose phase in which doses up to 36 mg were tested (*n* = 53), whereas Part B was a double-blind multiple-ascending-dose phase in which 9–36 mg of Y14 was administered at 7- to 14-day intervals over 28 days, with up to five doses per participant (*n* = 24). The PK profile supported this regimen. Predominant treatment-emergent adverse events were mild, self-limiting nausea, vomiting, and injection-site reactions, with no serious events. Exploratory outcomes showed placebo-adjusted weight loss of 2.87–3.58 kg at Day 31 and 38%–55% reductions in *ad libitum* food intake without tachyphylaxis [212].

## Conclusions and future directions

Recently approved GLP-1 agonist semaglutide and GLP-1/GIP dual agonist tirzepatide have redefined the therapeutic benchmark for metabolic diseases, including T2D, obesity, and MASLD/MASH. These agents have broadened diabetes care by achieving superior glycaemic control, substantial body-weight reduction, and cardio-renal-metabolic risk mitigations. Moreover, these incretin-based therapies have catalysed the development of a diverse pipeline of dual/multi-receptor agonists, demonstrating preliminary evidence of amplified clinical benefits. Here, we summarised recent updates on gut-pancreatic peptide signalling-targeted drug candidates that have received regulatory approval (Table 1) and those that remain in clinical development (Table 2).

**Table 1:** Regulatory approved gut-pancreatic peptide signalling-based therapies for metabolic disorders (as of April 2026)

Peptide therapy	Inventor/developer	Mechanism	Indications	Key clinical programmes	Approving regulatory authority
Liraglutide	Novo Nordisk	Long-acting GLP-1R agonist	T2D and Obesity	LEAD LEADER SCALE LEAN LIRAINS	FDA, EMA, TGA, and other agencies
Semaglutide	Novo Nordisk	Long-acting GLP-1R agonist	T2D, Obesity and non-cirrhotic MASH	SUSTAIN STEP PIONEER ESSENCE	FDA, EMA, TGA, and other agencies
Orforglipron	Eli Lilly and Company	Small-molecule GLP-1R full agonist	T2D and Obesity	ATTAIN ACHIEVE	FDA
Tirzepatide	Eli Lilly and Company	Dual incretin receptor agonist (GLP-1R/GIPR)	T2D and Obesity	SURPASS SURPASS-CVOT SURMOUNT	FDA, EMA, TGA, and other agencies
Pramlintide	AstraZeneca	Amylin analogue	Adjunct to insulin therapy in T1D and T2D	NCT00112021 NCT00673387 NCT00785408 NCT01235741 NCT00108004	FDA

**Table 2:** Summary of emerging gut-pancreatic peptide signalling-based therapies for metabolic disorders (as of April 2026)

Peptide therapy	Inventor/developer	Mechanism(s)	Primary indication(s)	Key clinical trials/programmes	Highest reported development
***GLP-1 analogues***					
Efpeglenatide	Hanmi Pharmaceutical and Sanofi	Long-acting GLP-1R agonist with reduced β-arrestin-2 recruitment	T2D	LIBERATE 204 BALANCE AMPLITUDE-M AMPLITUDE-O	Phase-3
Noiiglutide	Jiangsu Hengrui Pharmaceuticals	Long-acting GLP-1R agonist	T2D; Obesity	NCT06649773 NCT03848793 NCT04799327	Phase-3
TG103	CSPC Pharmaceutical Group	Long-acting GLP-1R agonist	T2D; Obesity	NCT05997576 NCT04855292 NCT03990090	Phase-3
Ecnoglutide	Sciwind Biosciences	Long-acting GLP-1R agonist with cAMP-biased signalling	T2D; Obesity	CTR20211014 NCT05680129	Phase-3
Utreglutide	Sun Pharma	Long-acting GLP-1R agonist with signalling bias favouring cAMP over β-arrestin-2 recruitment & receptor endocytosis	T2D; Obesity	EudraCT 2020-003765-20 NCT07282743 ACTRN12624000322538	Phase-2
***Oral GLP-1 formulations***					
Aleniglipron	Structure Therapeutics	Small-molecule GLP-1R agonist with biased agonism and reduced β-arrestin signalling	T2D; Obesity	NCT05762471 NCT06693843 NCT06703021	Phase-2
CT-996	Carmot Therapeutics Inc and Roche Ltd.	Small-molecule GLP-1R agonist with biased signalling	T2D; Obesity	NCT05814107	Phase-1
***GIPR agonist monotherapy***					
LY-3537021	Eli Lilly and Company	Long-acting selective GIPR agonist	T2D; Obesity	NCT04586907	Phase-1
***Dual agonists of GLP-1 and GIP***					
HS20094	Jiangsu Hansoh Pharmaceutical and Regeneron	Long-acting balanced dual GLP-1/GIPR agonist	T2D; Obesity	NCT05116410 NCT06118008 NCT06901648 NCT06118021 NCT06839664 NCT07156539	Phase-3
HRS9531	Jiangsu Hengrui Pharmaceuticals and Kailera Therapeutics	Long-acting full dual GLP-1/GIPR agonist	T2D; Obesity	NCT05152277 NCT05881837 NCT06054698 NCT05966272	Phase-3
VK2735	Viking Therapeutics	Long-acting full dual GLP-1/GIPR agonist	T2D; Obesity	VENTURE VANQUISH 1-2	Phase-3
CT-388	Carmot Therapeutics Inc and Roche	Long-acting biased dual GLP-1/GIPR agonist	T2D; Obesity	NCT04838405 NCT06628362 NCT06525935 NCT06628362	Phase-2
SCHO-094	SCOHIA Pharma	Long-acting full dual GLP-1/GIPR agonist	T2D; Obesity; MASH	EudraCT 2020-000999-37	Phase-1
**GIP antagonism-based therapies**					
MariTide	Amgen	Dual-acting GIPR antagonist and GLP-1R agonist	T2D; Obesity	NCT04478708 NCT05669599	Phase-3
**Glucagon agonist**					
Efpegerglucagon	Hanmi Pharmaceutical	Long-acting GCGR agonist	Congenital hyperinsulinism	NCT04032782 NCT04167553 NCT04732416	Phase-2
***Dual agonists of GLP-1 and glucagon***					
Survodutide	Boehringer Ingelheim and Zealand Pharma	Long-acting full agonist of GLP-1R and partial agonist of GCGR	T2D; Obesity; MASH	NCT04667377 NCT04153929 SYNCHRONIZE SYNCHRONIZE-CVOT LIVERAGE LIVERAGE-Cirrhosis	Phase-3
Mazdutide	Eli Lilly and Company	Long-acting dual GLP-1/GCGR agonist	T2D; Obesity	NCT04440345 NCT04466904 NCT04904913 NCT05607680	Phase-3
Pemvidutide	Altimmune Inc	Long-acting balanced dual GLP-1/GCGR agonist	Obesity; MASH	NCT04561245 NCT05006885 NCT05292911 NCT05295875 NCT05989711 NCT06987513	Phase-3
Efinopegdutide	Hanmi Pharmaceutical and Merck	Long-acting dual GLP-1/GCGR agonist	Obesity; MASH	NCT03486392 NCT03586830 NCT04944992 NCT05877547	Phase-2
Cotadutide	AstraZeneca	Unimolecular dual GLP-1/GCGR agonist	T2D; Obesity; MASH	NCT03244800 NCT04515849 NCT04019561	Phase-2
**Triple agonists of GLP-1, GIP, and glucagon**					
Retatrutide	Eli Lilly and Company	Long-acting triple GLP-1/GIP/GCGR agonist	T2D; Obesity	NCT04143802 NCT04881760 NCT04867785 NCT06260722 NCT05931367 NCT06859268 NCT07035093	Phase-3
Efocipegtrutide	Hanmi Pharmaceutical	Long-acting triple GLP-1/GIP/GCGR agonist	MASH	NCT03374241 NCT03744182 NCT04505436	Phase-2
**Amylin analogues/amylin-based combinations**					
Cagrilintide	Novo Nordisk	Long-acting amylin analogue with calcitonin receptor agonist activity	T2D; Obesity	NCT03600480 NCT03856047 NCT05567796	Phase-3
CagriSema	Novo Nordisk	Fixed-dose combination of cagrilintide (long-acting amylin/calcitonin receptor agonist) and semaglutide (GLP-1RA)	T2D; Obesity	NCT03600480 NCT04982575 NCT05567796 NCT05394519	Phase-3
Petrelintide	Zealand Pharma and Roche	Long-acting amylin analogue	Obesity	NCT05096598 NCT05613387 NCT06662539 NCT06926842	Phase-2
Amycretin	Novo Nordisk	Unimolecular long-acting GLP-1 and amylin receptor agonist	Obesity	NCT06064006 NCT05369390	Phase-2
Eloralintide	Eli Lilly and Company	Selective long-acting amylin receptor agonist	Obesity	NCT06230523	Phase-2
AZD6234	AstraZeneca	Long-acting selective amylin agonist	Obesity	NCT05511025	Phase-1
**PYY analogues**					
NNC0165-1875	Novo Nordisk	Long-acting PYY_3–36_ analogue	Obesity	NCT03707990 NCT04969939	Phase-2
NNC0165-1562	Novo Nordisk	Long-acting PYY analogue	Obesity	NCT02568306 NCT03574584	Phase-1
Y14 peptide	Imperial College London	Long-acting PYY analogue with high selectivity for neuropeptide Y2 receptors	Obesity	NCT03673111	Phase-1

At the conceptual level, mono-agonists act through a single dominant pathway, whereas dual and triple agonists are intended to exploit convergence at shared intracellular nodes, particularly cAMP-linked signalling pathways (Figure 2), while distributing their effects across the gut, brain, pancreas, adipose tissue, and liver. Whether multi-receptor targeting delivers true biological synergy, or mainly incremental efficacy, likely depends on receptor balance, tissue context, and chronic dosing, but this framework helps explain why some combinations achieve greater effects on body weight, glycaemic control, and liver fat than single-pathway approaches (Figure 2). In MASH-focused development programmes, reductions in hepatic steatosis have been more consistently demonstrated than fibrosis regression. This distinction has both clinical and regulatory relevance: early decreases in liver fat or aminotransferases may support therapeutic activity, but advanced MASH development is generally judged by histological endpoints such as MASH resolution without worsening of fibrosis and/or fibrosis improvement without worsening of MASH. Taken together, findings from semaglutide, tirzepatide, survodutide, pemvidutide, and retatrutide indicate that hepatic benefit frequently tracks with the extent of weight loss and overall metabolic improvement, whereas convincing evidence of direct weight-loss-independent anti-fibrotic activity remains incomplete.

The benchmark for next-generation anti-obesity therapeutics is no longer weight loss alone but durable and metabolically favourable weight reduction achieved with preservation of lean mass and meaningful improvement in obesity-related comorbidities. Most emerging metabolic hormone-based pharmacotherapies are derived from gut-hormone mimetics centred on GLP-1R agonism, frequently combined with co-activation of GIP, glucagon, amylin, or PYY pathways, and several now deliver mean weight reductions approaching or exceeding 15% to 20% in clinical studies. As efficacy increases, however, evaluation must extend beyond routine GI adverse events and instead focus on the determinants of long-term clinical value, including the partitioning of weight loss between fat and lean mass, the net cardiometabolic benefit, and the durability of response during sustained treatment. Although available evidence suggests preferential fat-mass reduction, robust body-composition data remain limited for most emerging agents. The weight regain observed after treatment withdrawal, as demonstrated in SURMOUNT-4, further underscores that obesity is a chronic relapsing disease for which pharmacotherapy will often need to be sustained rather than administered as a time-limited intervention.

Taken together, the investigational gut-pancreatic peptide signalling-based therapies discussed here hold considerable promise for expanding the current therapeutic armamentarium for clinical management of T2D, obesity, and metabolic liver disease. However, their translation into routine clinical practice will depend on successful completion of rigorous clinical trials, demonstration of long-term efficacy and safety, and navigation of stringent regulatory evaluation processes. If these challenges can be overcome, the next generation of gut-pancreatic peptide signalling-based therapeutics may not simply broaden the therapeutic armamentarium but fundamentally redefine the standard of care across the spectrum of metabolic disease by delivering sustained benefits in glycaemic control, weight reduction, hepatic and renal health, and cardiovascular outcomes.

## Open Access

Open access for this article was enabled by the participation of the University of Western Australia in an all-inclusive Read & Publish agreement with Portland Press and the Biochemical Society under an agreement with CAUL.

## Abbreviations

AC: adenylate cyclase
AMPK: AMP-activated protein kinase
AMYRs: amylin receptors
AP: area postrema
BMI: body mass index
CI: confidence interval
CNS: central nervous systems
CTR: calcitonin receptor
CV: cardiovascular
cAMP: cyclic adenosine monophosphate
DPP-IV: dipeptidyl peptidase-IV
EEC: enteroendocrine L-cells
EPAC: exchange protein directly activated by cAMP
FDA: the U.S. Food and Drug Administration
GI: gastrointestinal
GIP: glucose-dependent insulinotropic polypeptide
GIPR: chronic hyperglycaemia-induced down-regulation of GIP receptor
GLP-1: glucagon-like peptide-1
GLP-1RA: glucagon-like peptide-1 receptor agonist
GPCRs: G protein-coupled receptors
Gαs: G protein alpha-s
HR: Hazard ratio
IgG: immunoglobulin-G
MACE: major adverse CV events
MASH: metabolic dysfunction-associated steatohepatitis
MASLD: metabolic dysfunction-associated steatotic liver disease
NASH: non-alcoholic steatohepatitis
NTS: nucleus tractus solitarius
PD: pharmacodynamic
PEG: polyethylene glycol
PI3K/AKT: phosphoinositide 3-kinase/protein kinase B
PKA: protein kinase A
PK: pharmacokinetics
PYY: peptide YY
SNAC: sodium N-[8-(2-hydroxybenzoyl] amino) caprylate
T2D: type-2 diabetes
vs: versus
